# Tryptophan Metabolism and Aryl‐Hydrocarbon Receptor Agonists in the Gut Microbiome of People With Myalgic Encephalomyelitis/Chronic Fatigue Syndrome

**DOI:** 10.1002/mbo3.70333

**Published:** 2026-06-22

**Authors:** David J. Esteban, Brynn Conrad, Autumn Cullinan, Sharon Luong, Jason Albaum, Victoria Wilk

**Affiliations:** ^1^ Department of Biology Vassar College Poughkeepsie New York USA; ^2^ Independent Researcher Moscow Idaho USA

**Keywords:** aryl hydrocarbon receptor, microbiome, myalgic encephalomyelitis, tryptophan

## Abstract

Myalgic encephalomyelitis/chronic fatigue syndrome (ME/CFS) is a debilitating chronic disease with unknown biological basis and no cure. Microbiome dysbiosis has been reported in people with ME/CFS but its relevance to pathophysiology is unknown. Gut microbes are an important source of tryptophan metabolites that activate the aryl hydrocarbon receptor (AHR), a regulator of homeostatic and inflammatory genes. Dysregulated activation of AHR contributes to pathophysiology of several neuroimmune and chronic diseases but its role in ME/CFS has not been investigated. The purpose of this study was to investigate the production of tryptophan metabolites and AHR agonists by gut microbes of people with ME/CFS. We found lower diversity and altered microbiome community structure in people with ME/CFS and changes in the subcommunity of microbes that correlated with tryptophan metabolites. Using targeted metabolomics we identified nine metabolites elevated in the stool of people with ME/CFS, including three AHR agonists. Stool *ex vivo* cultures were tested for their capacity to activate AHR in a reporter cell line and by qPCR. AHR activation did not differ between people with ME/CFS and controls, however, we detected elevated agonist activity in people with neurocognitive symptoms, regardless of underlying disease. These findings are consistent with previous work revealing changes in the gut microbiome of people with ME/CFS and adds further support to alterations in tryptophan metabolism associated with the disease. Altered AHR activity by gut microbial metabolites may be a common mechanism contributing to neurocognitive symptoms in diseases including ME/CFS.

## Graphical abstract

The gut microbiome of people with myalgic encephalomyelitis/chronic fatigue syndrome (ME/CFS) is investigated to identify changes in microbial tryptophan metabolism. We reveal a subcommunity of microbes that correlates with tryptophan metabolism that differs in structure and diversity and identify differences in stool indoles including aryl‐hydrocarbon receptor agonists.

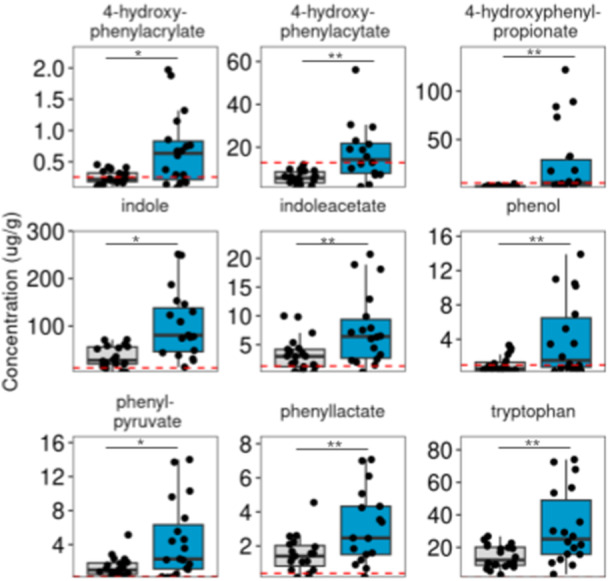

## 1 Introduction

Myalgic encephalomyelitis/chronic fatigue syndrome (ME/CFS) is a debilitating chronic disease diagnosed by its symptoms, which include post‐exertional malaise, cognitive dysfunction or “brain fog”, and orthostatic intolerance (Institute of Medicine of the National Academies 2015; Bateman et al. 2021). As a consequence of the debilitating symptoms, only 27‐ 43% of people with ME/CFS are employed (Rimbaut et al. 2016; Eaton‐Fitch et al. 2020), and 25% are largely unable to leave their home or bed (Pendergrast et al. 2016). Women are more likely to be diagnosed (Lim et al. 2020; Vahratian et al. 2023) and an estimated 1.5 million Americans are affected (Mirin et al. 2022), although this is likely an underestimate since about 58% of people with Long COVID meet ME/CFS diagnostic criteria (Jason and Dorri 2023).

The underlying basis of disease in ME/CFS is unknown, however several models have been proposed (Morris et al. 2016; Blomberg et al. 2018; Hatziagelaki et al. 2018; Proal and Marshall 2018; Kashi et al. 2019; Komaroff 2019). Many patients report onset following an infection, while others report trauma or injury (Chu et al. 2019), which may then lead to long term effects on multiple organ systems. Research has revealed measurable differences in people with ME/CFS including neuroinflammation, immunological dysregulation, metabolic alterations, and disrupted gastrointestinal homeostasis (VanElzakker et al. 2019; Mandarano et al. 2020; Mueller et al. 2020; Maya et al. 2023; Walitt et al. 2024), however no reliable biomarkers have yet been identified.

The dysregulation of these systems may be exacerbated by alterations in the gut microbiome. This complex and dynamic community of microorganisms affects homeostasis of the gastrointestinal, nervous, and immune systems, and connects to the brain via a circuit known as the microbiome‐gut‐brain axis (Cryan et al. 2019; Gheorghe et al. 2019). Several studies have shown disease‐associated alterations (termed dysbiosis) in the gut microbiome of people with ME/CFS (Frémont et al. 2013; Giloteaux et al. 2016; Nagy‐Szakal et al. 2017; Guo et al. 2023; He et al. 2023; Xiong et al. 2023).

Gut microbes are an important source of neuroactive and immunoregulatory metabolites, including tryptophan‐derived ligands of the aryl hydrocarbon receptor (AHR), a ligand‐activated nuclear receptor that regulates expression of genes tied to homeostasis, inflammation, and the response to xenobiotics (Stockinger et al. 2014; Neavin et al. 2018; Barroso et al. 2021). AHR is a key mediator of the microbiome‐gut‐brain axis. It is highly expressed in the gut, immune cells, microglia, and astrocytes, and has been shown to have effects on neuronal activity (Rothhammer et al. 2016, 2018; Rothhammer and Quintana 2019; Teng et al. 2022; Huang et al. 2023). Dysregulation of AHR and microbial tryptophan metabolism is implicated in pathogenesis of many neurological diseases, including multiple sclerosis (MS) (Quintana et al. 2008; Rothhammer et al. 2016, 2018), the neurological effects of chronic kidney disease (Pascussi et al. 2008; Watanabe et al. 2014; Liu et al. 2021; Bhargava et al. 2022; Huang et al. 2023), post‐stroke brain damage (Benakis et al. 2016; Singh et al. 2016; Wang et al. 2022), Alzheimer's disease (Salminen 2023), irritable bowel syndrome (IBS) (Berstad et al. 2014; Chojnacki et al. 2022; Meynier et al. 2022), and Long COVID (Guo et al. 2023).

No prior studies directly address AHR activation in ME/CFS, however several findings suggest potential for AHR involvement. People with ME/CFS show depletion of tryptophan synthesizing bacteria in the gut (Xiong et al. 2023) and lower plasma indole 3‐lactate, a partial AHR agonist (Nagy‐Szakal et al. 2018), while others have proposed an increased capacity for indole production and utilization of tryptophan by the gut microbiome (Dehhaghi et al. 2022). AHR balances inflammation and immune homeostasis through regulating development and activity of T_reg_ cells, Th17 cells (Quintana et al. 2008; Veldhoen et al. 2008) and mast cells (MC) (Sibilano et al. 2012; Zhou et al. 2013; Kawasaki et al. 2014) all of which are altered in ME/CFS (Curriu et al. 2013; Smylie et al. 2013; Brenu et al. 2013; Hornig et al. 2017; Rivas et al. 2018; Sepúlveda et al. 2019; Simonato et al. 2021). Cathelicidin and TGF‐ β, which were elevated in studies of ME/CFS plasma (Corbitt et al. 2019; Milivojevic et al. 2020), together can activate AHR‐dependent proinflammatory Th17 differentiation (Minns et al. 2021). Dysregulation of the host kynurenine pathway, which also produces AHR agonists (Dinatale et al. 2010; Seok et al. 2018; Vyhlídalová et al. 2020), may also be important in pathophysiology of ME/CFS (Anderson et al. 2012; Morris et al. 2016; Kashi et al. 2019; Ormstad et al. 2020; Dehhaghi et al. 2022; Kavyani et al. 2022).

Dysregulation of AHR activation is consistent with the gastrointestinal symptoms, dysbiosis, neurological, and immunological alterations in ME/CFS and is consistent with proposed models of the disease. The purpose of this study was to investigate the production of tryptophan metabolites including AHR agonists by gut microbes in people with ME/CFS.

## 2 Methods and Materials

### 2.1 Participant Selection and Surveys

Use of human subjects was approved by the Vassar College IRB and the Solve ME/CFS Initiative IRB (WCG IRB Protocol # 1271974). Participants were recruited and consented through the Solve ME/CFS Initiative You+ME patient registry (renamed SolveTogether). Exclusion criteria included having taken oral or intravenous antibiotics less than 4 weeks prior, a COVID‐19 diagnosis or quarantine less than 4 weeks prior, residing outside the US, pregnancy, age under 18 years, and no signed consent form. Participants of any gender were included, and cases required illness lasting at least 3 years. Cases required an ME/CFS diagnosis that met Institute of Medicine (IOM), Canadian Consensus Criteria (CCC), or CDC criteria. Assessment was based on patient‐completed responses to the UK ME/CFS Biobank (UKMEB) symptom assessment questionnaire, which determines fulfilment of distinct ME/CFS criteria of all the major clinical definitions and is scored using an algorithm licensed to the You+ME Registry by the UKMEB (Lacerda et al. 2018; Ramiller et al. 2022). Controls did not have an ME/CFS diagnosis and were excluded if they met clinical criteria for ME/CFS based on symptom survey responses. Cases and controls were sex and age (+/‐ 5 years) matched. All eligible participants completed additional surveys in the You+ME registry (Ramiller et al. 2022), including demographics, medical history, symptom assessment, Karnofsky scale (Mor et al. 1984), and SF‐12 (Ware et al. 1996).

### 2.2 Statistical Analysis of Survey Data

Demographics of the case and control participants were analyzed using Fisher's exact test, except body mass index (BMI) for which we used a Wilcoxon Rank Sum test. Karnofsky Performance Scores, SF‐12 Mental Component Summary (MCS) score, and SF‐12 Physical Component Summary score (PCS) of cases and controls were compared using a Wilcoxon Rank Sum test.

### 2.3 Stool Sample Collection

Participants were provided with an at‐home stool sample collection kit (EZ Sampler, ALPCO), which included a collection tube with scoop, a flushable collection tray, neoprene gloves, and packaging material. Participants were advised not to allow urine or toilet water to come in contact with the sample. Samples were to be shipped immediately, or stored in the fridge and shipped the next day in a cold shipping box with a push‐button activated cooling pack that provides 48 h cooling during shipping. Samples received were immediately placed at 4°C until processing the same day or next day. No preservative solution was used during collection or shipping because samples were used for *ex vivo* culturing.

### 2.4 Stool Sample Processing and Ex Vivo Stool Cultures

Within 1 day of receiving the sample, 1 g stool was added to 9 mL sterile saline (0.85% NaCl) and vortexed horizontally for at least 10 min to homogenize. 0.5 mL of the suspension was used for cell counts and 1 mL of the suspension was used for *ex vivo* cultures.

*Ex vivo* cultures were prepared with 1 mL stool suspension in 9 mL Stool Culture Medium (SCM; 1% glucose, 50 mM Na2HPO4, pH 7.4, with or without 1 mM tryptophan), then incubated for 24 h at 37°C with shaking. Following incubation, the culture was centrifuged at 15,000 g for 15 min, the supernatant was transferred to a new centrifuge tube and centrifuged a second time. Supernatant was then filtered using a 0.2 μm syringe filter and stored at −80°C.

### 2.5 Cell Counts

A 0.5 ml aliquot of saline stool suspension was centrifuged at 10,000 g for 5 min, and the pellet resuspended in 1 mL sterile water to wash. The centrifugation and water wash was repeated a total of three times. Ten‐fold serial dilutions of resuspended pellet to 1:1000 were prepared and 90 μL of sample was mixed with 0.67 μL BacLight bacterial viability stain (Invitrogen, MA) followed by incubation at room temp in the dark for at least 5 min. Stained sample was mixed with an equal volume of ProLong Gold mounting medium (Invitrogen, MA) and placed on a slide with coverslip. Slides were imaged on a fluorescence microscope (Nikon Eclipse E400) at 400X magnification obtaining 10 random field images of each slide for live (excitation 470/40, emission 525/50) or dead (excitation 540/25, emission 605/55). Spot analysis was carried out using Fiji software to count live and dead cells. Cell counts were analyzed in R using a linear model on log transformed data using demographics, stool property (texture and storage time) and disease status.

### 2.6 16S rRNA Gene Sequencing

DNA was extracted from approximately 0.25 g stool using a Power Fecal DNA Extraction Kit (Qiagen, Germantown, MD) and used to prepare 16S rRNA gene amplicon libraries. Amplification of the V3‐V4 region was performed using 5 μL of each universal primer at 1 μM

(Forward 5′‐TCGTCGGCAGCGTCAGATGTGTATAAGAGACAGCCTACGGGNGGCWGCAG‐3′ reverse 5′‐GTCTCGTGGGCTCGGAGATGTGTATAAGAGACAGGACTACHVGGGTAT CTAATCC‐3′), 12.5 μL of 2X KAPA HiFi Hot Start Ready Mix (Kapa Biosystems), 1 μL template DNA, and 1.5ul water. Thermocycling conditions were as follows: 95°C for 3 min, 30 cycles of 95°C for 30 s, 55°C for 30 s, 72°C for 30 s, and a final step 72°C for 5 min. Product size was confirmed by gel electrophoresis and the product was purified using a QIAquick PCR Purification Kit (Qiagen). Next, a PCR was performed to attach Illumina index primers (IDT for Illumina DNA/RNA UD Indexes Set A, Illumina Inc, San Diego, CA). Each reaction contained 12.5 μL of 2X KAPA HiFi Hot Start Ready Mix, 5 μL of index primers, 5 μL nuclease‐free water, and 2.5 μL of the purified first‐stage PCR product. Thermocycling conditions were as follows: 95°C for 3 min, 8 cycles of 95°C for 30 s, 55°C for 30 s, 72°C for 30 s, and a final step 72°C for 5 min. A QIAquick PCR Purification Kit was used to purify indexed PCR products and concentration was determined using a NanoDrop One (ThermoFisher, Pittsburgh, PA). Each product was diluted to 4 nM in 1 M Tris pH 8.0 prior to pooling all samples at 1 nM. Immediately before sequencing, a final dilution to 75 pM was prepared and 5% v/v 75 pM PhiX control (Illumina) was added. The pool was sequenced using an Illumina iSeq 100 with reagent cartridge v2.

### 2.7 Microbiome Analysis

Reads were processed using QIIME2‐amplicon‐2024.2 (Bolyen et al. 2019). Forward reads were denoised and clustered at 100% identity (Amplicon Sequence Variants, ASV) using Dada2 with default settings (Callahan et al. 2016). ASVs with a total frequency less than 4 or that appeared in only one sample were removed. Taxonomy was assigned to ASVs using *qiime feature‐classifier classify‐sklearn* with a SILVA‐99 classifier prepared specifically for taxonomic assignment of iSeq 100 generated sequences, followed by removal of chloroplast, mitochondrial, and sequences that could not be classified at the taxonomic level of Kingdom. A rooted tree was generated for phylogenetic diversity analyses using the q2‐phylogeny plugin, which uses mafft (Katoh and Standley 2013) and fasttree (Price et al. 2010). Rarefaction was performed to determine the appropriate sampling depth for further diversity analysis. Subsequent analyses were performed using 10500 sequences per sample to calculate Shannon index (Shannon 1948), Pielou evenness (Pielou 1966), dominance, and Faith's phylogenetic diversity (Faith 1992).

Shannon index, Pielou evenness, dominance, and Faith's phylogenetic diversity were tested for normality using quantile‐quantile (QQ) plots and a Shapiro‐Wilk test. Faith's phylogenetic diversity data were not normally distributed however log transformation, which resulted in a normal distribution, gave similar results in linear models so untransformed data were used.

Statistical analysis was performed in R v.3.5. 2 (R Core Team 2020). Linear models were used to analyze alpha and beta diversity as response variables. Predictors in full models included disease status, stool sample storage time (days from collection to sample processing), stool sample texture (categorized as firm, soft, or runny), and demographic variables (age, sex, BMI, income, and disability status). These explanatory variables were chosen because of known effects of these variables on the gut microbiome, or differences between our patient and control groups. Other demographic variables were excluded because of a lack of variability. Inclusion of demographic variables resulted in a smaller sample size for analysis due to some incomplete survey responses. Simplified models constructed through backwards elimination of non‐significant predictors (*p* > 0.05) were also constructed and compared to full models using AIC and are presented in supporting materials. Where full models resulted in significant predictors but an overall non‐significant model, we present the simplified model with the best fit. Impacts of specific symptoms were assessed in separate linear models that included disease status (ME/CFS or control), presence of IBS symptoms, neurocognitive symptoms, and stool sample texture and storage time.

Beta diversity analyses (weighted and unweighted UniFrac (Lozupone et al. 2010), Bray‐Curtis index (Bray and Curtis 1957), and Jaccard index (Real and Vargas 1996) and PCoA were calculated in qiime2. PCoA plots were generated using the ggplot2 package (Wickham 2016). Permutational multivariate analysis of variance (Permanova) with 999 permutations was performed using the same explanatory predictors described above in *adonis2*, and homogeneity of variances (dispersion) for disease status was tested using *betadispr*, both from the R package *vegan* (Oksanen et al. 2020). Firmicutes:Bacteroidetes was analyzed using log transformed data and a linear model including demographic, stool property, and disease status as predictors.

### 2.8 Gene Expression Quantification (qPCR)

Caco‐2 cells were maintained in minimal essential medium (MEM) with Earle's salts and l‐glutamine (Gibco, MA) supplemented with 10% newborn calf serum (NBCS, Gibco), 1X non‐essential amino acids (NEAA, Gibco) and 1X penicillin/streptomycin (Cytiva, UT). Following trypsinization, cells were counted using a Bright‐Line hemacytometer, diluted to 1 × 10^5^ cells/mL, and 1 mL per well was cultured in 24‐well plates for 24 h at 37°C and 5% CO_2_ to allow attachment. Media was then removed and replaced with 0.5 mL media containing 2.5 μM FICZ (6‐formylindolo[3,2‐b] carbazole; InvivoGen) as a positive control, media alone as a negative control, or 1:16 dilution of supernatant from *ex vivo* cultured stool samples. After 6 h of incubation at 37°C and 5% CO_2_, RNA was extracted from cells (Pure‐Link RNA Mini Kit, Invitrogen, CA). Cells were lysed in 300 μL lysis buffer and passed through an 18‐gauge needle prior to processing through the extraction kit. RNA was quantified using a NanoDrop ONE spectrophotometer (ThermoFisher, WI).

RT‐PCR was performed to synthesize cDNA using Superscript III First‐Strand synthesis kit (Invitrogen, CA) with 1 μL 50 mM oligo‐dT primer, 1 μL annealing buffer, and 6 μL RNA. Samples were incubated at 65°C for 5 min then transferred to ice before adding 10 μL 2X First Strand reaction mix and 2 μL reverse transcriptase enzyme mix, then placed in a thermocycler at 50°C for 50 min followed by 85°C for 5 min. cDNA concentrations were measured on a NanoDrop ONE and diluted to 50 ng/μL. qPCR was used to measure expression of AHR inducible genes CYP1A1 and AHRR and housekeeping gene GAPDH. Each reaction contained 10 μL TaqMan Fast Advanced Mastermix (Applied Biosystems, MA), 1 μL GAPDH probe (Hs 99999905_m1 VIC, Applied Biosystems), 1 μL target gene probe (CYP1A1 Hs01054796_g1 FAM or AHRR Hs01005075_m1 FAM, Applied Biosystems), 6 μL nuclease free water and 2 μL cDNA, and was cycled in a QuantStudio3 Real Time PCR system (AppliedBiosystems, MA) using the following conditions: 50°C for 2 min, and 40 cycles of 95°C for 2 min, 95°C for 1 s, 60°C for 20 s. Data was analyzed using the 2–∆∆Ct method. Values were normalized by cultured sample weight. Initial complex linear models included sample storage time (time from collection to processing), disease status (ME/CFS or control), and presence of neurocognitive symptoms. Linear models with neurocognitive symptoms, disease status (ME/CFS vs. control), and stool storage time were used for AHRR and CYP1A1 fold expression change.

### 2.9 AHR Reporter Assay

H1L6.1c3 cells (Van Langenhove et al. 2011) were generously provided by Jan‐Peter van Pijkeren (University of Wisconsin, Madison). Cells were maintained in DMEM‐10 (Dulbecco's Modified Eagle Medium with 10% newborn calf serum (Gibco), 1% penicillin/streptomycin (Cytiva), and 1% l‐glutamine (Gibco). Cells were grown to near confluence, harvested with 0.25% trypsin‐EDTA, and counted on a Bright‐Line Hemacytometer (Hausser Scientific). A volume of 200 µL cell suspension at 3 × 10^5^ cells/ml was added to black‐walled clear‐bottom 96‐well tissue culture plates (Costar, ME) and incubated at 37°C and 5% CO_2_ for 48 h to allow attachment. Afterward, media was removed from all wells and replaced with 100 µL/well of treatment or controls. The positive control FICZ (InvivoGen) was diluted in DMEM‐10 to 0.05 µM, negative controls used DMEM‐10 only. *Ex vivo* stool culture supernatants were diluted 1:10 in DMEM‐10. The plate was then incubated at 37°C and 5% CO_2_ for 2 h. Viability was determined by adding 20 µL per well of CellTiter‐Fluor reagent (Promega, WI) followed by incubation at 37°C and 5% CO_2_ for 30 min then fluorescence was measured using a BioTek Synergy HTX Multi‐Mode Microplate Reader using 360/40 nm excitation and 528/20 nm emission wavelengths. Luciferase activity was measured using ONE‐Glo Luciferase Assay System (Promega). 100 µL/well of luciferase reagent was added to each well and incubated at room temperature for 10 min and luminescence measured on the plate reader. Luciferase activity was normalized by viability, and both were normalized to initial stool sample weight cultured. After removal of outliers by quartile method and checks for normality, the data were analyzed by linear models using predictors disease status, neurocognitive symptoms, and storage time of stool sample prior to culturing.

### 2.10 Metabolomics

Frozen stool samples from 42 participants (*N* = 19 ME/CFS, *N* = 23 Controls) were shipped to Precion Metabolomics (Morrisville, NC) for targeted analysis of 77 metabolites, which were prepared following Precion's Microbiome Specific Metabolite Panel assay. A solution of stable isotope labeled internal standards was added to samples followed by simultaneous homogenization, protein precipitation and extraction. The extracts were analyzed with four different LC‐MS/MS methods specific to the analytes.

Data analysis was performed using MetaboAnalyst 6.0 (Pang et al. 2024). Metabolite concentrations were normalized by stool dry weight, outliers were removed using the 1.5 interquartile range method, and twelve metabolites without quantifiable values in at least 50% of samples were removed from further analysis. An additional four low variance metabolites were removed by filtering the bottom 5% of metabolites with the lowest variance based on interquartile range. Missing values were imputed using ⅕ of the minimum positive value of that metabolite. These filtering steps were performed without consideration of disease or other grouping, and resulted in 61 metabolites retained for analysis. To identify metabolites that differed significantly between cases and controls, or among different reported symptoms, FDR corrected t‐tests with *p* < 0.05 and fold change threshold of 2 were used. Subsequent multivariate linear models of those significant metabolites were constructed in R to include demographic and stool sample properties (disease, age, sex, BMI, income, disability status, stool texture, and stool sample storage time). Shapiro‐Wilk tests and QQ plots were used to check for normality and non‐normal data were log transformed.

To investigate the relationship between microbes and tryptophan metabolites, a spearman correlation of tryptophan metabolite concentrations and ASV abundance was performed in R. We first removed low abundance ASVs by selecting only those that made up at least 0.01% of the total relative abundance. A total of seven tryptophan metabolites were selected for correlation analysis that were significantly different (*p* < 0.05) between ME/CFS and controls before FDR correction in wet‐weight or dry‐weight normalized samples. Microbiome analysis of the tryptophan metabolite‐correlated subcommunity, which included only ASVs that correlated with at least one tryptophan metabolite, was performed as described above for the whole community, except for alpha diversity analysis the samples were rarefied to 5000 sequences per sample.

## 3 Results

### 3.1 Participants

We received samples from 67 participants (35 controls, 32 cases). Participants did not differ in any demographic measures except that significantly more cases were on disability (Fisher's exact test, *p* = 0.047) and more controls were from higher income families (Fisher's exact test, *p* = 0.019), (Table 1). Although ME/CFS is overrepresented in women, we had approximately equal numbers of male and female participants. Our study sample was primarily white and non‐Hispanic.

**Table 1:** Demographics of study population.

Feature	ME/CFS	Control	P^a^
Participants (n)	32	35	NA
Age (mean)	50.1	51.1	0.801
Female (%)	47.8	52.2	0.749
Mean BMI	25.8	24.7	0.226
Military (%)	6.3	8.6	1.00
Current smoker (%)	3.1	5.7	1.00
**Race and Ethnicity (%)**			
Non‐Hispanic	79.4	94.3	0.246
Race:White	87.5	89.2	1.00
Race:Black	0	0	NA
Race:Asian	6.3	5.4	0.603
Race:Hawaii/PI	0	0	NA
Race:Indigenous/Native American	3.1	2.7	0.478
Race:Not Reported	3.1	2.7	1.00
**Education (%)**			
High school	0	2.9	NA
Some college	15.6	2.9	0.099
College	40.6	28.6	0.436
Graduate degree	40.6	60.0	0.132
**Work Status (%)**			
Employed/student/homemaker	25	48.6	0.082
Unemployed	15.6	25.7	0.376
Retired	3.1	5.7	1.00
On disability	12.5	0	**0.047**
**Income**			
Income: <$100 K	40.6	40.0	1.00
Income: >$100 K	18.8	45.7	**0.019**

*Note:* Values in bold *p* < 0.05.

^a^ Fisher's exact test, except BMI Wilcoxon rank sum test.

**Figure 1:**
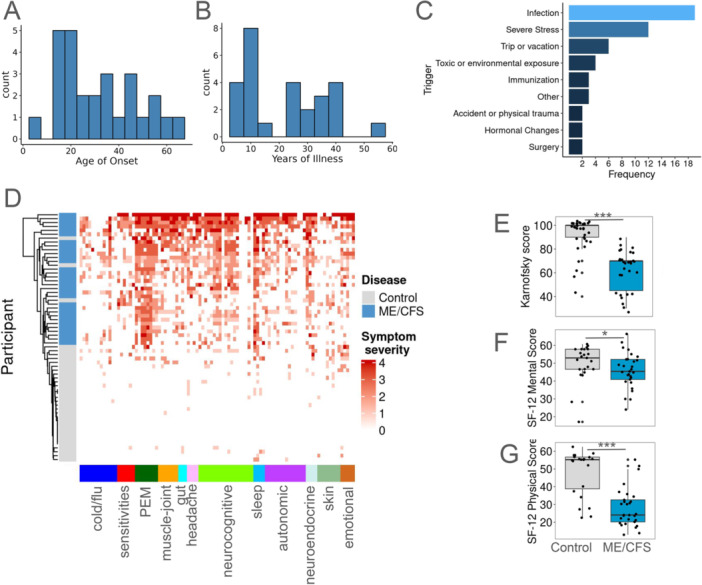
Characteristics of the ME/CFS cases and controls. Participants completed surveys using the Solve ME/CFS You+ME Registry. Not all participants completed every survey question resulting in smaller sample sizes for certain questions. (A) Frequency of reported age of ME/CFS onset (*n* = 27 ME/CFS). (B) Frequency of duration of illness (n = 27 ME/CFS). (C) Frequency of most commonly self‐reported disease triggers (*n* = 29 ME/CFS); more than one trigger was reported for many participants. (D) Heatmap of participant reported symptoms and their severity experienced within the 2 weeks prior to completing the survey. Each column represents the response to a survey question, categorized by system affected below the plot. Severity of symptoms is shown by the intensity of the red in the plot. Participants in rows were organized by clustering dendrogram shown at left with the disease status shown. (E) Karnofsky Performance Score, which measures functional impairment (*n* = 33 control, 30 ME/CFS). For participants who completed the SF‐12 survey (*n* = 23 controls, 29 ME/CFS), the mental and physical component scores were calculated. (F) Mental Component Summary score of the SF‐12 survey, measuring the impact of mental health on functioning, (G) Physical Component Summary score of the SF‐12 survey, measuring the impact of physical health on functioning. ***p < 0.001, *p < 0.05.

Based on symptom survey responses, all ME/CFS cases met CCC and/or IOM criteria except one which met CDC criteria. Age of disease onset ranged from childhood to over 60 years (Figure 1A), and duration of illness ranged from 5 to 57 years (Figure 1B). Infection was the most common reported trigger (Figure 1C). For symptoms experienced within the 2 weeks prior to completing the survey, participants were asked to rank the severity of symptoms, and the results are presented in a heatmap in Figure 1D. As expected based on the diagnostic criteria, patients demonstrate post exertional malaise (PEM), as well as sleep‐related symptoms, neurocognitive and neuroendocrine symptoms, and emotional (mental health) symptoms. Less common were skin symptoms, cold or flu‐like symptoms, and sensitivities (such as to chemicals, medications, or foods). Cases scored significantly lower on the Karnofsky performance scale (Figure 1E) (Wilcoxon rank sum test: *W* = 903, *P* = 9.80 × 10^−9^), and significantly lower on the SF‐12 physical score (Wilcoxon rank sum test: *W* = 585, P = 3.75 × 10^−^^6^) and mental score (Wilcoxon rank sum test: *W* = 445, *p* = 0.001), (Figure 1F,G) as expected for this highly debilitating disease.

### 3.2 Stool Microbiome

**Figure 2:**
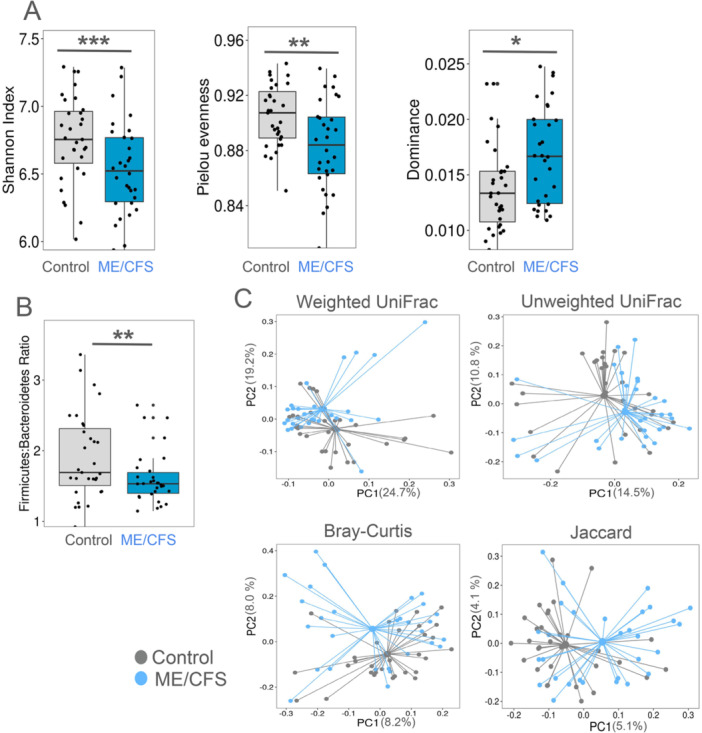
Dysbiosis in the microbiome of people with ME/CFS. 16S rRNA gene sequencing was performed to characterize the structure and diversity of the microbial community. (A) Community diversity was assessed with Shannon index, Pielou evenness, and dominance. Linear models revealed a significant effect of disease on diversity (***p < 0.001, **p < 0.01, *p < 0.05). (B) Ratio of the abundance of Firmicutes to Bacteroidetes, a commonly used measure of dysbiosis (**p < 0.01). (C) Differences in community structure were evaluated with beta diversity metrics shown in PCoA plots and the effect of disease on community structure was tested using permanovas. Weighted UniFrac (*p* < 0.01), Unweighted Unifrac (*p* < 0.01), Bray‐Curtis (*p* < 0.05), and Jaccard index (*p* < 0.01).

To characterize the stool microbiome community, we used 16S rRNA gene sequencing and analyzed the community structure and diversity using various measures of alpha and beta diversity (Figure 2A). There were 61 samples (*n* = 31 controls and *n* = 30 ME/CFS) with sufficient sequencing depth for the variables tested in diversity analyses.

We used linear models to determine the effect of disease, demographics, and stool sample properties on diversity and community structure. Models included sample storage time (days between collection and processing), stool texture (firm, soft, or runny), demographic variables likely to influence microbiomes (age, sex, bmi) and demographic variables that differed between our control and ME/CFS participants (income and disability status), which may impact the microbiome through socioeconomic factors like diet and differential access to healthcare. Because the linear models require complete data for analysis and some demographic data was missing, the number of samples used in the analysis was fewer for the full models (*N* = 28 controls, *N* = 26 ME/CFS). Details of the contribution of each explanatory variable as well as simplified models generated through backwards elimination of non‐significant predictors are available in Table A1 for alpha diversity and Table A2 for beta diversity metrics.

For the Shannon index, disease status, age, sex, and stool storage time were significant predictors (LM: *F*_9,44_ = 3.98, *p* = 0.00090, adj *R* ^2^ = 0.34) with lower diversity in ME/CFS microbiomes. Disease status was a significant predictor of Pielou evenness however the full model was non‐significant (LM: *F*_9,44_ = 1.35, *p* = 0.24, adj *R* ^2^ = 0.06). A simplified model using only disease status demonstrated a better fit by AIC, showing disease status had an effect on evenness, albeit with a low proportion of variance explained (LM: *F*_1,58_ = 10.05, *p* = 0.0024 adj *R* ^2^ = 0.13). Disease status and age were significant predictors of dominance, the relative abundance of the most abundant amplicon sequence variant (ASV) (LM: *F*_9,44_ = 2.56, *p* = 0.018, adj *R* ^2^ = 0.21). Faith's phylogenetic diversity was not affected by any of the test variables (LM: *F*_9,44_ = 1.42, *p* = 0.21, adj *R* ^2^ = 0.068). In summary, Shannon index, evenness, and dominance showed a consistent effect of disease, while storage time, age and sex affected certain diversity metrics but not others.

Given the higher dominance in microbiomes of people with ME/CFS, we next investigated the identity of the dominant taxa. Microbiomes of people with ME/CFS were more often dominated by members of the phylum Bacteroidetes than Firmicutes so we calculated the Firmicutes:Bacteroidetes ratio (Figure 2B) and performed a linear model using disease, demographics, and stool properties as above. The full model identified disease and storage time as the only significant predictors but was a poor fit compared to a simplified model including only disease and storage time as a predictors. Using the simplified model, the Firmicutes:Bacteroidetes ratio, with people with ME/CFS having a significantly lower Firmicutes:Bacteroidetes ratio than controls (*F*_2,57_ = 5.48, *p* = 0.0067, adj *R* ^2^ = 0.13).

We next compared the microbiome structure of people with ME/CFS and controls using beta diversity metrics (Figure 2C) and Permanova using the same variables as above. We report here the statistical values for significant predictors in the full models; details of all predictors as well as simplified models using backwards elimination are available in Table A2. On community structure as measured by Bray‐Curtis dissimilarity, which incorporates species richness and abundance, there was a significant effect of disease (Permanova: pseudo‐F_56_ = 1.39, *p* = 0.036, *R* ^2^ = 0.024), age (Permanonva: pseudo‐F_56_ = 1.79, *p* = 0.001, *R* ^2^ = 0.03), storage time (Permanova: pseudo‐F_56_ = 1.56, *p* = 0.007, *R* ^2^ = 0.026), and stool texture (Permanova: pseudo‐F_56_ = 1.51, *p* = 0.006, *R* ^2^ = 0.051). For the Jaccard index, which incorporates species richness but not abundance (only presence or absence) there was a significant effect of disease (Permanova: pseudo‐F_56_ = 1.25, *p* = 0.009, *R* ^2^ = 0.022), age (Permanova: pseudo‐F_56_ = 1.39, *p* = 0.001, *R* ^2^ = 0.023) and storage time (Permanoa: pseudo‐F_56_ = 1.33, *p* = 0.003, *R* ^2^ = 0.023). There was also a significant effect of disease (Permanova: pseudo‐F_56_ = 2.88, *p* = 0.005, *R* ^2^ = 0.047) and storage time (Permanova: pseudo‐F_56_ = 2.66, *p* = 0.016, *R* ^2^ = 0.044) on weighted UniFrac, which incorporates phylogenetic diversity and abundance. Unweighted UniFrac, which incorporates phylogenetic diversity and presence/absence (no abundance) was significantly affected by disease (Permanova: pseudo‐F_56_ = 2.88, *p* = 0.009, *R* ^2^ = 0.047) and storage time (Permanova: pseudo‐F_56_ = 2.67, *p* = 0.013, *R* ^2^ = 0.044). Dispersion of Bray‐Curtis dissimilarity was affected by disease status (ANOVA: *F*_59_ = 4.49, *p* = 0.038) however other metrics did not differ in dispersion based on disease (All Anovas: F_59_ < 3.86, *p* > 0.05) In summary, these metrics reveal a consistent, albeit small, effect of disease on community structure. Storage time, the time between collection and processing, also consistently affected community structure.

Finally, we determined the number and ratio of live and dead bacterial cells from stool samples using fluorescence microscopy. There was no effect of disease on live cell count (LM: *F*_8_,_39_ = 1.42, *p* = 0.22, *R* ^2^ = 0.07) or dead cell count (LM: *F*_8,39_ = 1.22, *p* = 0.27, *R* ^2^ = 0.06).

### 3.3 Metabolomics

**Figure 3:**
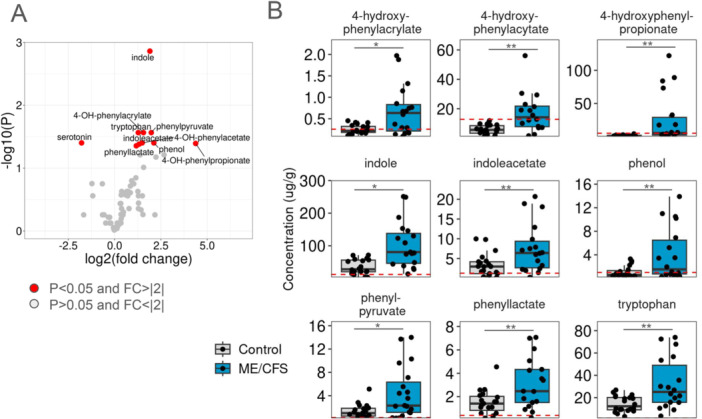
Aromatic amino acid metabolite concentrations in stool differ between people with ME/CFS and controls. Targeted metabolomics was performed on stool samples (*n* = 23 Control, 19 ME/CFS). 67 metabolites were detected in at least 50% of samples. (A) Volcano plot to identify metabolites with fold changes >|2| and FDR corrected statistical significance (*p* < 0.05). (B) Metabolite concentrations in ME/CFS cases and controls. Linear models revealed a significant effect of disease (*p < 0.05, **p < 0.01). Dashed red lines show the threshold of detection for each metabolite.

We performed targeted metabolomics of stool samples to quantify 77 metabolites, including 16 microbial products of tryptophan metabolism. Twelve metabolites were detected in fewer than 50% of samples and were removed from analysis and another four were removed due to low variance. To determine if stool samples from people with ME/CFS differed in metabolite concentrations from those of controls, we first generated a volcano plot to visualize fold change and FDR adjusted *P*‐value from *t*‐tests of metabolite concentrations normalized to dry weight of stool (Figure 3A). Ten metabolites showed a fold change > |2| and FDR corrected *p*‐value < 0.05. Of these, serotonin was dropped from further analysis because all values fell below the lower limit of quantification. We further analyzed the remaining nine metabolites using linear models of log transformed data. Linear models included disease, stool properties, and demographic predictors as used above (see Table A3 for model details). For all metabolites tested, except phenyllactate, disease status was a significant predictor of disease in the full model (all LM: *F*_9,27_ > 2.4, *p* < 0.05, adj *R* ^2^ > 0.26), with concentrations elevated in stool samples of people with ME/CFS (Figure 3B). For phenyllactate, disease status was a significant predictor in an overall non‐significant model; a simplified model using backwards elimination resulted in a better fit with disease and disability status as significant predictors (LM: *F*_2,33_ = 6.80, *p* = 0.0034, adj *R* ^2^ = 0.25). The predictors income, disability, age, sex, stool sample storage time, and stool texture were significant for at most three different metabolites, making disease status the only consistent predictor with a significant effect on all metabolite concentrations. Notably, indole, indole acetate, and tryptophan are all AHR agonists. The others are tyrosine or phenylalanine metabolites, together indicating alteration of aromatic amino acid metabolism in the stool of people with ME/CFS.

**Figure 4:**
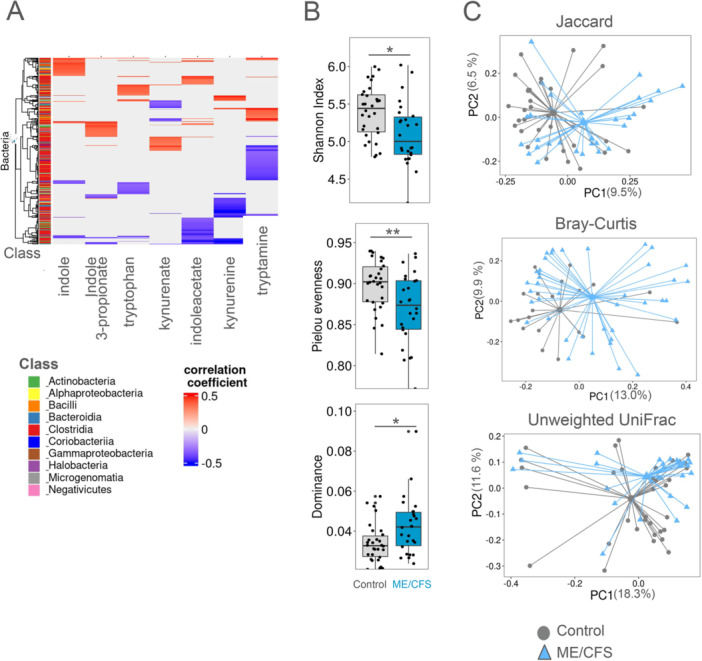
Tryptophan metabolite correlated bacterial subcommunity differs between people with ME/CFS and controls. Tryptophan metabolites with concentrations that differed between ME/CFS and controls prior to FDR correction and bacteria that were present in at least 0.1% relative abundance were selected for analysis. (A) Heatmap of Spearman correlation of metabolites and bacterial abundances, only those bacteria that correlated significantly with at least one metabolite are shown. Bacteria were clustered by a dendrogram, and taxonomic Class is shown in the bars to the left of the heatmap. **(**B) Alpha diversity metrics of the subset of the microbial community that correlates with tryptophan metabolites show that this subcommunity is less diverse in people with ME/CFS (*p < 0.05, **p < 0.01). (C) Beta diversity metrics of the subset of the microbial community that correlates with tryptophan metabolites. Bray‐Curtis (*p* < 0.01), unweighted UniFrac (*p* < 0.05), and Jaccard index (*p* < 0.01) revealed that the structure of this subcommunity differed between ME/CFS and controls (Permanova).

Because we were interested in the role of tryptophan metabolism and AHR agonists in ME/CFS, we performed a Spearman correlation analysis to identify relationships between bacterial relative abundance and metabolite concentrations. We identified 373 ASVs with a significant correlation to at least one tryptophan metabolite (Figure 4A). The majority were Clostridia, a Class known to be important producers of metabolites that affect host physiology. Although some bacteria correlated both positively or negatively with different tryptophan metabolites, most species consistently showed only negative or only positive correlations with the metabolites.

This may be a functionally relevant subcommunity given the role of tryptophan metabolites in host physiology, so we sought to determine if the tryptophan metabolite correlated microbial subcommunity differed between people with ME/CFS and controls. Alpha diversity metrics revealed lower diversity in this subcommunity in people with ME/CFS (Figure 4B) (LM: Dominance: *F*_9,44_ = 2.20, *p* = 0.041, adj *R* ^2^ = 0.17; Shannon index: *F*_9,44_ = 3.161, *p* = 0.0005, adj *R* ^2^ = 0.27) using linear models that also included demographic and stool properties (Full model details are available in Table A4). A linear model of Pielou evenness with all predictors revealed disease as the only significant predictor in an overall non‐significant model, so a simplified model constructed by backwards elimination to include disease status had a significant effect on evenness (LM: *F*_2,53_ = 5.062, *p* = 0.0097, adj *R* ^2^ = 0.13). Linear models of Faith's phylogenetic diversity again showed no effect from disease status. No other predictor was consistently significant in measures of alpha diversity.

To investigate changes in the structure of the tryptophan metabolite correlated subcommunity, we used permanovas with demographic, stool properties and disease status as explanatory variables and report here the effect of disease status on beta diversity measures; details of other variables are given in Table A5. People with ME/CFS and controls differed in the structure of this sub‐community by three beta diversity metrics (Figure 4B) (Permanova: Bray Curtis: pseudo‐F_58_ = 1.94, *p* = 0.007, *R* ^2^ = 0.03; Unweighted UniFrac: pseudo‐F_58_ = 1.76, *p* = 0.043, *R* ^2^ = 0.027; Jaccard: pseudo‐F_58_ = 1.55, *p* = 0.009, *R* ^2^ = 0.025), while weighted UniFrac was not affected by disease (Permanova: pseudo‐F_58_ = 1.75, *p* = 0.092, *R* ^2^ = 0.027). Stool texture and storage time also significantly affected Bray Curtis and Jaccard index (Table A5). There was no difference in dispersion by disease status, indicating a shift in the location of the centroids explains the differences (ANOVA: Bray Curtis: *F*_1,61_ = 1.16, *p* = 0.29; Jaccard: *F*_1,61_ = 0.24, *p* = 0.62; unweighted UniFrac: *F* = 1.11, *p* = 0.30). Thus, the dysbiosis seen in people with ME/CFS includes lower diversity and a shift in the subcommunity of bacteria that may be involved in tryptophan metabolism.

### 3.4 AHR Activation and Neurocognitive Symptoms

**Figure 5:**
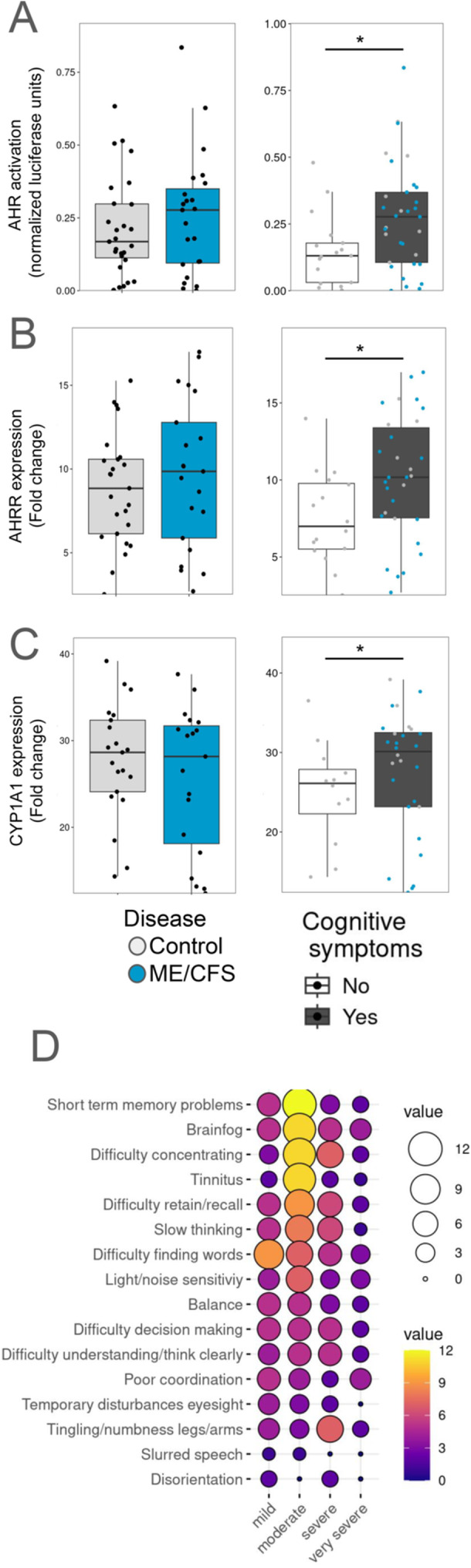
AHR agonist activity in *ex vivo* stool cultures is elevated in people with neurocognitive symptoms. (A) H1L6.1c3 cells expressing luciferase under control of AHR were treated with *ex vivo* stool culture supernatants. Luminescence was normalized to cell viability. Linear models revealed an effect of presence of neurocognitive symptoms (*n* = 17 no symptoms, 33 with symptoms) on AHR agonist activity but not disease (*n* = 27 controls, 23 ME/CFS). Caco2 intestinal epithelial cells were treated with *ex vivo* stool culture supernatants and expression of AHR regulated genes AHRR (B) or CYP1A1 (C) were evaluated by TaqMan qPCR (*n* = 16 no symptoms, 30 with symptoms). GAPDH was used as an internal control. Linear models revealed AHRR and CYP1A1 expression were elevated in cells treated with samples from individuals with neurocognitive symptoms but there was no effect of disease (*n* = 25 control, 21 ME/CFS). (D) Presence of neurocognitive symptoms was determined from symptom survey responses. The balloon plot shows the frequency of participants reporting each symptom and its severity. *p < 0.05.

We next sought to test the capacity of microbial communities from stool to activate AHR. We applied *ex vivo* stool culture supernatants to a reporter cell line that expresses luciferase under control of AHR‐regulated dioxin response elements (DREs) to evaluate the microbiome's capacity to produce AHR agonists. Culture supernatants were found to effectively induce AHR‐mediated luciferase expression similar to a positive control agonist, FICZ, and have no effect on cell viability. We used linear models with disease status, neurocognitive symptoms, and stool storage time as predictors. We present below the results for each predictor. We found no difference in AHR agonist activity between people with ME/CFS and controls (LM: *F*_3,46_ = 0.62, *p* = 0.42) (Figure 5A). We also tested AHR agonist activity in stool *ex vivo* culture supernatants by measuring expression of two canonical AHR‐regulated genes, CYP1A1 and AHRR, in an intestinal epithelial cell line (Caco2) by qPCR. There was no difference in induction of AHRR (LM: *F*_3,42_ = 0.64 *p* = 0.43) or CYP1A1 (LM: *F*_3,42_ = 0.95, *p* = 0.34) expression by *ex vivo* stool culture supernatants from people with ME/CFS and controls (Figure 5B,C). We were unable to detect AHR agonist activity in either assay using uncultured homogenized and saline‐suspended stool samples.

AHR agonists are important mediators of the gut‐brain axis and neuroinflammation, therefore we also investigated whether there was a relationship between AHR agonist activity and the presence of neurocognitive symptoms. All participants with ME/CFS reported neurocognitive symptoms, but because some of the control participants also reported neurocognitive symptoms due to different underlying conditions, we grouped all participants with neurocognitive symptoms together regardless of underlying disease (Figure 5D). We found that those who reported neurocognitive symptoms showed elevated AHR agonist activity in the luciferase reporter cell line (Figure 5A) (LM: *F*_3,46_ = 5.68, *p* = 0.022). In treated Caco2 cells, we detected elevated AHRR expression (LM: *F*_3,42_ = 5.38, *p* = 0.025) and elevated CYP1A1 expression (LM: *F*_44_ = 4.32, *p* = 0.045). There was no effect of stool sample storage time on AHR activity in any assay.

### 3.5 Symptoms and the Microbiome

Presence of IBS symptoms can affect the microbiome, and given the differences in AHR agonist activity in *ex vivo* stool cultures based on neurocognitive symptoms, we investigated the impact of these disease symptoms on diversity and community structure. Linear models included disease status (ME/CFS vs. Control), presence of IBS symptoms, presence of neurocognitive symptoms, as well as stool texture and storage time (which were relevant predictors in many other models). Presence of IBS or neurocognitive symptoms had no effect on measures of alpha diversity, while ME/CFS disease remained a significant predictor for Shannon index and dominance (Table A6) Similarly, presence of IBS or neurocognitive symptoms had no effect on measures of beta diversity, while ME/CFS disease remained a significant predictor (Table A7).

Finally, we investigated differences in stool metabolite concentrations. There were no differences in metabolite concentrations of participants with or without neurocognitive symptoms. However, prior to FDR correction, three metabolites were elevated in stool samples from participants with neurocognitive symptoms: indole (*p* = 0.0096, *t* = 2.72), indoleacetate (*p* = 0.019, *t* = 2.44), and 4‐hydroxyphenylacetate (*p* = 0.040, *t* = 2.12). Enterolactone was significantly lower in stool samples of participants with neurocognitive symptoms (*p* = 0.0011, *t* = 3.54).

Taken together, these results suggests that the differences in the microbiome and metabolome are specific to disease status and not IBS or neurocognitive symptoms, and that elevated AHR agonist activity in people with neurocognitive symptoms is a consequence of differences in active metabolism of supplemental tryptophan by microbes in the community during *ex vivo* culture rather than pre‐existing differences in the community prior to culturing.

## 4 Discussion

Gut microbiome dysbiosis in people with ME/CFS has been reported in several studies (Giloteaux et al. 2016; Nagy‐Szakal et al. 2017; Kitami et al. 2020; Lupo et al. 2021; Guo et al. 2023; Xiong et al. 2023), and the importance of the bidirectional communication between host and microbiome to the regulation of host physiology underscores the need to investigate the role of dysbiosis in disease. In this study, we sought to expand our understanding of dysbiosis by investigating microbial tryptophan metabolism, with a particular interest in indoles that are AHR agonists.

We confirmed dysbiosis with several diversity metrics that revealed shifts in microbial community structure and lower diversity in our sample of people with ME/CFS, adding further evidence of changes in the microbiomes of people with ME/CFS. While the differences are small, shifts in microbiome communities as a consequence of disease that is not primarily gastrointestinal are often subtle and our results are in line with other studies in ME/CFS (Guo et al. 2023). In addition, both alpha and beta diversity metrics consistently revealed an effect of disease in both the whole community and the tryptophan metabolite‐correlated subcommunity, increasing our confidence that the modest effect size is nonetheless important. Gut microbiomes are affected by disease but also numerous other variables, including lifestyle factors like diet, socioeconomic status, and medications (Aasmets et al. 2025). In our study, disease remained a significant factor in shaping the microbiome even after adjusting for several demographic covariates. However, we were not able to include diet and medications in our analyses, both of which are likely to vary between patient and control groups.

Using targeted metabolomics of fecal samples, we identified elevated tryptophan and some of its metabolites, as well as several metabolites of tyrosine and phenylalanine indicating changes in aromatic amino acid metabolism. We also revealed shifts in the subcommunity of tryptophan metabolizers, heavily comprised of members of the Clostridiales. These differences may have functional consequences for regulation of host physiology. Tryptophan, indole, and indole 3‐acetate are agonists of AHR, a nuclear receptor implicated in pathogenesis of other neuroimmune diseases.

Several metabolomics and gut metagenomics studies have been performed on people with ME/CFS. Guo et al (Guo et al. 2023) found depletion of butyrate and sulfate metabolism genes in the gut metagenome, with enrichment in several other metabolic pathways in patients, and reduced short chain fatty acid (butyrate and acetate) concentrations in the feces. While our metabolite panel included several short chain fatty acids, we detected no differences between groups. Guo et al (Guo et al. 2023) found no difference in tryptophan metabolic pathway representation in the metagenome but did find depletion of trp metabolizing bacteria by taxonomy. Only a few other studies have investigated the metabolites in stool of people with ME/CFS. Lupo (Lupo et al. 2021) performed untargeted metabolomics on stool of ME/CFS patients and found some differences. Notably, indole was not in their set of annotated compounds. Dehhagi et al (Dehhaghi et al. 2022) predicted an increased capacity for indole production and utilization of tryptophan by the gut microbiome, consistent with our findings. In addition, several studies have revealed differences in plasma metabolites, including lower plasma indole 3‐lactate, a partial AHR agonist (Nagy‐Szakal et al. 2018) and a mix of enriched and depleted tryptophan metabolites (Abujrais et al. 2024). Inconsistent results among studies may be attributable to differences among participant populations due to clinical diagnostic criteria, including prevalence of PEM, and the diverse study techniques from metagenomics, targeted or untargeted metabolomics, amplicon sequencing, and functional assays, each of which reveal different aspects of the microbial community. The relationship between fecal metabolites and plasma or serum metabolites in people with ME/CFS needs further investigation.

When stool samples were cultured with tryptophan to measure the community's potential to produce AHR agonists when provided tryptophan, we found no difference between stool from people with ME/CFS and controls, however we detected elevated agonist activity associated with the presence of neurocognitive symptoms, which were also reported among some controls. Most of the symptoms reported in this category are related to cognition, however others, like sensitivity to noise or light, indicate this cluster of symptoms may be more broadly related to neuroinflammation. While this finding does not reveal a feature specific to ME/CFS disease, it nonetheless may be important to people with ME/CFS, because “brain fog” and other cognitive difficulties are common and debilitating symptoms for people with ME/CFS. This underscores the relevance and potential benefit of understanding the relationship between neurocognitive symptoms and gut dysbiosis regardless of whether it is a unique feature of the disease or common to people experiencing neurocognitive symptoms more broadly. Further, we did not identify differences in microbial community diversity or structure based on presence or absence of neurocognitive symptoms, instead the difference is revealed as a consequence of tryptophan supplementation during culturing, suggesting a different response, rather than a different community, is driving this difference.

Dysregulation of AHR and microbial tryptophan metabolism is implicated in pathogenesis of many neurological diseases. In the mouse model of multiple sclerosis (MS) knockout of AHR in astrocytes or microglia resulted in worsening of disease due to elevated neuroinflammation, and IFN‐B induced expression of astrocyte AHR helps control neuroinflammation by limiting NF‐kB mediated pro‐inflammatory gene expression (Rothhammer et al. 2016, 2018). Indole 3‐sulfate, produced in the liver from indole produced by gut microbes, acts through AHR to drive neurological effects of chronic kidney disease (Pascussi et al. 2008; Watanabe et al. 2014; Liu et al. 2021; Bhargava et al. 2022; Huang et al. 2023). In IBS, which is typically accompanied with some cognitive and mood symptoms, one study found that acute tryptophan depletion lowered blood tryptophan and kynurene concentrations and eliminated the cognitive performance deficit in patients (Kennedy et al. 2014). Animal models also support a role for gut microbial AHR agonists in IBS related neurological and cognitive symptoms (Meynier et al. 2022). In Long COVID, astrocyte dysfunction involving genes regulated by AHR may contribute to “brain fog” (Horowitz et al. 2023). Therefore it is possible that common underlying AHR‐driven mechanisms contribute to neuroinflammation in a variety of diseases, and ME/CFS may be included among them. Because our study included some control participants that reported neurocognitive symptoms, we were only able to detect this signal when participants were grouped by symptom, not disease, perhaps due to this underlying commonality. Future studies using quantitative measures of cognitive performance, such as memory and attention tasks, will be necessary to better understand the contribution of AHR agonists to disease symptoms of ME/CFS.

In our study we also identified elevated tryptophan metabolites in the stool of people with ME/CFS. The relationship between stool and blood concentrations of tryptophan metabolites is not clear, however even local changes to tryptophan metabolites, including AHR agonists, may have systemic effects due to their role in regulating intestinal permeability and mucosal immune networks. Prior research implicates a disrupted balance of tryptophan metabolism and AHR agonists in ME/CFS. AHR regulates development of anti‐inflammatory *T*_reg_ cells and pro‐inflammatory Th17 cells (Quintana et al. 2008; Veldhoen et al. 2008) which are altered in ME/CFS (Curriu et al. 2013; Smylie et al. 2013; Brenu et al. 2013; Hornig et al. 2015; Rivas et al. 2018; Sepúlveda et al. 2019; Simonato et al. 2021). Cathelicidin and TGF‐ β, which are elevated in the plasma of people with ME/CFS (Corbitt et al. 2019; Milivojevic et al. 2020) induce AHR‐dependent Th17 differentiation (Minns et al. 2021). Mast cells (MC), which are abundant in the gut and can contribute to inflammation in the brain, express AHR (Sibilano et al. 2012; Zhou et al. 2013; Kawasaki et al. 2014) and MC Activation Syndrome is a common comorbidity in people with ME/CFS (Hatziagelaki et al. 2018). AHR also regulates gut permeability in response to environmental and gut microbiome signals, and “leaky gut” has been reported in ME/CFS (Shukla et al. 2015; Giloteaux et al. 2016). In addition, dysregulation of the cellular kynurenine pathway, which produces several AHR agonists from tryptophan, is important in pathophysiology of ME/CFS (Morris et al. 2017; Kashi et al. 2019; Dehhaghi et al. 2022; Kavyani et al. 2022). Simonato (Simonato et al. 2021) found lower concentrations of kynurenine, elevated 3‐hydroxykynurenine, and elevated kynurenic acid:kynurenine ratio, together suggesting increased transformation of kynurenine. Others have identified IDO1 mutations in people with ME/CFS and proposed a model in which a shift in the balance of the kynurenine pathway contributes to underlying disease(Kashi et al. 2019). Together, these findings and those reported in this study, reveal altered tryptophan metabolism in both the host and microbiome that may be a contributor to disrupted homeostasis in ME/CFS.

Our results must be considered in the context of the limitations of the study. Participants were assigned status as ME/CFS or controls based on self‐report and a symptom survey. The survey is used by UK ME/CFS Biobank and SolveME/CFS Initiative, and participants are scored using an algorithm based on clinical criteria for diagnosis. However, our participants were not evaluated by study physician. Given the variation in presentation of the disease and the absence of a clinical biomarker, this approach can lead to a more heterogenous study sample. All participants had a long duration of illness, thus where other studies have revealed differences between early and late stages of disease (Hornig et al. 2015; Xiong et al. 2023) we were unable to evaluate the effect of disease duration on our results. We also recruited a study population that was evenly balanced between males and females, and primarily white, although the disease affects a greater proportion of women and affects people of all races. All symptoms were self‐reported, including neurocognitive symptoms and cognitive function. Future studies should make use of validated cognitive tests and measures of neuroinflammation to better characterize the relationship between elevated AHR activity and neurocognitive symptoms. Microbiome differences in the subsets of people with ME/CFS with or without IBS has also been shown (Nagy‐Szakal et al. 2017; Guo et al. 2023) however in our study we did not find an effect of gut symptoms. It is possible that based on our symptom surveys we were not able to as effectively identify people with IBS to reveal these disease subgroups or that our study is insufficiently powered to detect disease subgroups. Stool samples were collected and shipped without addition of stabilizers or other preservatives. Several studies report minimal or no impact of this approach on the microbiome (Wasfy et al. 1995; Nel et al. 2020; Plauzolles et al. 2022). For our study, this approach was necessary to allow *ex vivo* culturing, however, we did find that time between collection and processing (storage time) had an effect on the community. Nonetheless, even considering the effect of storage time, ME/CFS disease status remained a significant explanatory variable for community structure and diversity.

Further investigation into the role of tryptophan metabolism and AHR in ME/CFS may reveal a path to therapeutic intervention targeting cognitive or neurological symptoms. There are approved and experimental drugs that are AHR agonists (Safe et al. 2017; Smith et al. 2017; Fakan et al. 2019) and dietary intervention with tryptophan depletion has been shown to reduce cognitive symptoms in people with IBS (Kennedy et al. 2015) thus presenting different avenues for intervention. However, given that AHR balances inflammation and immune homeostasis, and activation of AHR is pathogenic in some diseases but protective in others, the way forward for ME/CFS is not yet clear. By revealing differences in AHR agonists and activation, this work identifies a relevant area for further investigation.

## Author Contributions

**David J. Esteban:** conceptualization, funding acquisition, writing – original draft, methodology, visualization, writing – review and editing, formal analysis, project administration, supervision, investigation, data curation, validation, resources. **Brynn Conrad:** investigation, writing – review and editing, formal analysis, validation. **Autumn Cullinan:** investigation, methodology, formal analysis, writing – review and editing, validation. **Sharon Luong:** investigation, methodology, writing – review and editing, formal analysis, validation. **Jason Albaum:** investigation, methodology, writing – review and editing, validation. **Victoria Wilk:** formal analysis, writing – review and editing.

## Ethics Statement

The protocol and procedures employed were reviewed and approved by the Vassar College IRB and the Solve ME/CFS Initiative IRB (WCG IRB Protocol # 1271974).

## Conflicts of Interest

The authors declare no coflicts of interest.

## Supporting information


**Table A1:** Alpha diversity models with whole microbiome.

**Table A2:** Beta diversity models with whole microbiome.

**Table A3:** Metabolites models.

**Table A4:** Alpha diversity models with Trp correlated subcommunity.

**Table A5:** Beta diversity models with Trp correlated subcommunity.

**Table A6:** Alpha diversity symptom models.

**Table A7:** Beta diversity symptom models.

## Data Availability

DNA sequence data has been deposited in NCBI SRA BioProject with the accession number PRJNA1332076. All data and analysis files including R code and QIIME2 commands, can be found on FigShare with identifier 10.6084/m9. figshare.30278434. The data that support the findings of this study are openly available in NCBI SRA BioProject at https://www.ncbi.nlm.nih.gov/sra, reference number PRJNA1332076.
