# Supplementary material for: Tryptophan Metabolism and Aryl‐Hydrocarbon Receptor Agonists in the Gut Microbiome of People With Myalgic Encephalomyelitis/Chronic Fatigue Syndrome
Source: Microbiologyopen. 2026 Jun 22;15(3):e70333. doi: 10.1002/mbo3.70333 (PMC13284739; doi:10.1002/mbo3.70333)
Supplement: Supplementary file 1 — Table A1: Alpha diversity models with whole microbiome. [file MBO3-15-e70333-s003.docx]

|  |  |  |  |  |  |  |  |  |
| --- | --- | --- | --- | --- | --- | --- | --- | --- |
| DISEASE (MECFS vs Controls), DEMOGRAPHICS, STOOL PROPERTIES MODELS with WHOLE MICROBIOME | | | | | | | | |
|  |  |  |  |  |  |  |  |  |
| Full Models | Shannon index** | |  |  | Faith's PD (log transformed) | | |  |
|  | Estimate | Std. Error | t | P | Estimate | Std. Error | t | P |
| (Intercept) | 6.299 | 0.318 | 19.827 | <2e-16 | 2.455 | 0.401 | 6.117 | 0.000 |
| Disease(MECFS) | -0.230 | 0.086 | -2.679 | **0.010** | -0.103 | 0.063 | -1.627 | 0.111 |
| demog_age | 0.010 | 0.003 | 3.855 | **0.000** | 0.004 | 0.002 | 2.010 | 0.051 |
| demog_sex | 0.216 | 0.080 | 2.690 | **0.010** | 0.097 | 0.057 | 1.710 | 0.094 |
| demog_bmi | -0.022 | 0.012 | -1.877 | 0.067 | -0.063 | 0.106 | -0.596 | 0.555 |
| demog_incomeunder100K | 0.116 | 0.083 | 1.404 | 0.167 | 0.109 | 0.108 | 1.011 | 0.318 |
| demog_work_ondisab | -0.134 | 0.131 | -1.020 | 0.313 | -0.001 | 0.009 | -0.143 | 0.887 |
| texturerunny | 0.051 | 0.222 | 0.228 | 0.821 | 0.088 | 0.065 | 1.364 | 0.180 |
| texturesoft | 0.101 | 0.090 | 1.118 | 0.270 | 0.003 | 0.045 | 0.077 | 0.939 |
| processing time | 0.072 | 0.027 | 2.658 | **0.011** | 0.022 | 0.093 | 0.237 | 0.814 |
|  | p-value: 0.0008958 | |  |  | p-value: 0.206 | |  |  |
|  | Adjusted R-squared: 0.3362 | | |  | Adjusted R-squared: 0.06768 | | | |
|  | F-statistic: 3.983 on 9 and 44 DF, | | | | F-statistic: 1.427 on 9 and 44 DF | | | |
|  |  |  |  |  |  |  |  |  |
| Full Models | Pielou evenness | |  |  | Dominance | |  |  |
|  | Estimate | Std. Error | t | P | Estimate | Std. Error | t | P |
| (Intercept) | 0.931 | 0.030 | 30.737 | <2e-16 | 0.017 | 0.004 | 3.745 | 0.001 |
| Disease(MECFS) | -0.020 | 0.008 | -2.478 | **0.017** | 0.003 | 0.001 | 2.637 | **0.012** |
| demog_age | 0.000 | 0.000 | -0.337 | 0.738 | 0.000 | 0.000 | -2.352 | **0.023** |
| demog_sex | 0.001 | 0.008 | 0.072 | 0.943 | -0.002 | 0.001 | -1.714 | 0.094 |
| demog_bmi | 0.000 | 0.001 | -0.334 | 0.740 | 0.000 | 0.000 | 1.395 | 0.170 |
| demog_incomeunder100K | 0.003 | 0.008 | 0.430 | 0.670 | -0.002 | 0.001 | -1.425 | 0.161 |
| demog_work_ondisab | -0.006 | 0.013 | -0.499 | 0.620 | 0.002 | 0.002 | 1.147 | 0.258 |
| texturerunny | -0.015 | 0.021 | -0.701 | 0.487 | 0.000 | 0.003 | -0.059 | 0.953 |
| texturesoft | -0.002 | 0.009 | -0.244 | 0.809 | -0.001 | 0.001 | -0.810 | 0.422 |
| processing time | -0.003 | 0.003 | -1.222 | 0.228 | -0.001 | 0.000 | -1.385 | 0.173 |
|  | p-value: 0.2419 | |  |  | p-value: | 0.018 |  |  |
|  | Adjusted R-squared: 0.05547 | | | | Adjusted R-squared: 0.2095 | | |  |
|  | F-statistic: 1.346 on 9 and 44 DF | | | | F-statistic: 2.561 on 9 and 44 DF | | | |
|  |  |  |  |  |  |  |  |  |
|  |  |  |  |  |  |  |  |  |
| Simplified Models* | Shannon index | |  |  | Faith's PD | |  |  |
|  | Estimate | Std. Error | t | P | Estimate | Std. Error | t | P |
| (Intercept) | 6.166 | 0.198 | 31.090 | <2e-16 | no simplified model constructed | | | |
| Disease(MECFS) | -0.225 | 0.082 | -2.730 | **0.009** |  |  |  |  |
| demog_age | 0.007 | 0.003 | 2.598 | **0.012** |  |  |  |  |
| processing_time | 0.059 | 0.026 | 2.291 | **0.026** |  |  |  |  |
|  |  |  |  |  |  |  |  |  |
|  | p-value: 0.001813 | |  |  |  |  |  |  |
|  | Adjusted R-squared: 0.1953 | | |  |  |  |  |  |
|  | F-statistic: 5.693 on 3 and 55 DF | | | |  |  |  |  |
|  |  |  |  |  |  |  |  |  |
| Simplified Models* | Pielou evenness** | |  |  | Dominance** | |  |  |
|  | Estimate | Std. Error | t | P | Estimate | Std. Error | t | P |
| (Intercept) | 0.905 | 0.005 | 182.228 | 0.000 | 0.014 | 0.001 | 18.637 | 0.000 |
| Disease(MECFS) | -0.023 | 0.007 | -3.171 | **0.002** | 0.003 | 0.001 | 2.951 | **0.005** |
| demog_age |  |  |  |  |  |  |  |  |
| processing_time |  |  |  |  |  |  |  |  |
|  | p-value: | 0.002 |  |  | p-value: | 0.005 |  |  |
|  | Adjusted R-squared: 0.133 | | |  | Adjusted R-squared: 0.1155 | | |  |
|  | F-statistic: 10.05 on 1 and 58 DF | | | | F-statistic: 8.707 on 1 and 58 DF | | | |
|  |  |  |  |  |  |  |  |  |
| *constructed through iterative backwards elimination of non-significnat (P>0.05) variables in the full model | | | | | | | | |
| **model performs best by AIC | |  |  |  |  |  |  |  |
