# Supplementary material for: Tryptophan Metabolism and Aryl‐Hydrocarbon Receptor Agonists in the Gut Microbiome of People With Myalgic Encephalomyelitis/Chronic Fatigue Syndrome
Source: Microbiologyopen. 2026 Jun 22;15(3):e70333. doi: 10.1002/mbo3.70333 (PMC13284739; doi:10.1002/mbo3.70333)
Supplement: Supplementary file 2 — Table A2: Beta diversity models with whole microbiome. [file MBO3-15-e70333-s007.docx]

|  |  |  |  |  |  |  |  |  |  |  |  |
| --- | --- | --- | --- | --- | --- | --- | --- | --- | --- | --- | --- |
| DISEASE (MECFS vs Control) DEOGRAPHICS, STOOL PROPERTIES MODELS with WHOLE MICROBIOME | | | | | | | | | | |  |
| Full Models |  |  |  |  |  |  |  |  |  |  |  |
| Bray Curtis |  |  |  |  |  | Jaccard |  |  |  |  |  |
|  | Df | SumOfSqs | R2 | pseudo-F | Pr(>F) |  | Df | SumOfSqs | R2 | pseudo-F | Pr(>F) |
| Disease | 1 | 0.360 | 0.024 | 1.391 | **0.036** | Disease | 1 | 0.437 | 0.022 | 1.248 | **0.009** |
| age | 1 | 0.465 | 0.030 | 1.794 | **0.001** | age | 1 | 0.487 | 0.024 | 1.391 | **0.001** |
| sex | 1 | 0.278 | 0.018 | 1.073 | 0.301 | sex | 1 | 0.378 | 0.019 | 1.079 | 0.170 |
| bmi | 1 | 0.300 | 0.020 | 1.158 | 0.189 | bmi | 1 | 0.367 | 0.018 | 1.048 | 0.281 |
| income | 1 | 0.229 | 0.015 | 0.885 | 0.702 | disab | 1 | 0.349 | 0.017 | 0.996 | 0.454 |
| disab | 1 | 0.222 | 0.015 | 0.858 | 0.761 | income | 1 | 0.342 | 0.017 | 0.976 | 0.581 |
| texture | 2 | 0.783 | 0.051 | 1.513 | **0.006** | texture | 2 | 0.752 | 0.038 | 1.073 | 0.116 |
| storage | 1 | 0.403 | 0.026 | 1.555 | **0.007** | storage | 1 | 0.468 | 0.023 | 1.334 | **0.003** |
| Residual | 47 | 12.168 | 0.794 |  |  | Residual | 47 | 16.470 | 0.821 |  |  |
| Total | 56 | 15.319 | 1.000 |  |  | Total | 56 | 20.059 | 1.000 |  |  |
|  |  |  |  |  |  |  |  |  |  |  |  |
| Weighted UniFrac | |  |  |  |  | Unweighted UniFrac | | |  |  |  |
|  | Df | SumOfSqs | R2 | pseudo-F | Pr(>F) |  | Df | SumOfSqs | R2 | pseudo-F | Pr(>F) |
| Disease | 1 | 0.090 | 0.047 | 2.879 | **0.005** | Disease | 1 | 0.090 | 0.047 | 2.879 | **0.009** |
| age | 1 | 0.059 | 0.031 | 1.881 | 0.060 | age | 1 | 0.059 | 0.031 | 1.881 | 0.063 |
| sex | 1 | 0.025 | 0.013 | 0.814 | 0.599 | sex | 1 | 0.025 | 0.013 | 0.814 | 0.592 |
| bmi | 1 | 0.040 | 0.021 | 1.272 | 0.225 | bmi | 1 | 0.040 | 0.021 | 1.272 | 0.250 |
| disab | 1 | 0.023 | 0.012 | 0.724 | 0.627 | disab | 1 | 0.023 | 0.012 | 0.724 | 0.668 |
| income | 1 | 0.026 | 0.013 | 0.826 | 0.490 | income | 1 | 0.026 | 0.013 | 0.826 | 0.514 |
| texture | 2 | 0.064 | 0.033 | 1.022 | 0.374 | texture | 2 | 0.064 | 0.033 | 1.022 | 0.369 |
| storage | 1 | 0.083 | 0.044 | 2.668 | **0.016** | storage | 1 | 0.083 | 0.044 | 2.668 | **0.013** |
| Residual | 47 | 1.463 | 0.766 |  |  | Residual | 47 | 1.463 | 0.766 |  |  |
| Total | 56 | 1.909 | 1.000 |  |  | Total | 56 | 1.909 | 1.000 |  |  |
|  |  |  |  |  |  |  |  |  |  |  |  |
|  |  |  |  |  |  |  |  |  |  |  |  |
| Simplified models* | | |  |  |  |  |  |  |  |  |  |
| Bray Curtis |  |  |  |  |  | Jaccard |  |  |  |  |  |
|  | Df | SumOfSqs | R2 | pseudo-F | Pr(>F) |  | Df | SumOfSqs | R2 | pseudo-F | Pr(>F) |
| Disease | 1 | 0.429 | 0.027 | 1.674 | **0.003** | Disease | 1 | 0.489 | 0.023 | 1.391 | 0.002 |
| age | 1 | 0.434 | 0.027 | 1.691 | **0.002** | age | 1 | 0.461 | 0.022 | 1.309 | 0.006 |
| texture | 2 | 0.816 | 0.051 | 1.590 | **0.003** | storage | 1 | 0.477 | 0.023 | 1.356 | 0.002 |
| storage | 1 | 0.405 | 0.025 | 1.580 | **0.016** | Residual | 56 | 19.695 | 0.934 |  |  |
| Residual | 54 | 13.849 | 0.867 |  |  | Total | 59 | 21.091 | 1.000 |  |  |
| Total | 59 | 15.974 | 1.000 |  |  |  |  |  |  |  |  |
|  |  |  |  |  |  |  |  |  |  |  |  |
| Weighted UniFrac | |  |  |  |  | Unweighted UniFrac | | |  |  |  |
|  | Df | SumOfSqs | R2 | pseudo-F | Pr(>F) |  | Df | SumOfSqs | R2 | pseudo-F | Pr(>F) |
| Disease | 1 | 0.101 | 0.051 | 3.222 | **0.003** | Disease | 1 | 0.101 | 0.051 | 3.222 | **0.002** |
| storage | 1 | 0.081 | 0.041 | 2.580 | **0.009** | storage | 1 | 0.081 | 0.041 | 2.580 | **0.015** |
| Residual | 57 | 1.790 | 0.908 |  |  | Residual | 57 | 1.790 | 0.908 |  |  |
| Total | 59 | 1.972 | 1.000 |  |  | Total | 59 | 1.972 | 1.000 |  |  |
|  |  |  |  |  |  |  |  |  |  |  |  |
|  |  |  |  |  |  |  |  |  |  |  |  |
|  |  |  |  |  |  |  |  |  |  |  |  |
|  |  |  |  |  |  |  |  |  |  |  |  |
| * constructed by backwards elimination of nonsignificant (P>0.05) variables from full model | | | | | | | | | |  |  |
