# Supplementary material for: Tryptophan Metabolism and Aryl‐Hydrocarbon Receptor Agonists in the Gut Microbiome of People With Myalgic Encephalomyelitis/Chronic Fatigue Syndrome
Source: Microbiologyopen. 2026 Jun 22;15(3):e70333. doi: 10.1002/mbo3.70333 (PMC13284739; doi:10.1002/mbo3.70333)
Supplement: Supplementary file 3 — Table A3: Metabolites models. [file MBO3-15-e70333-s001.docx]

| Full Models |  |  |  |  |  |  |  |  |  |  |
| --- | --- | --- | --- | --- | --- | --- | --- | --- | --- | --- |
| Tryptophan |  |  |  |  |  | Indole |  |  |  |  |
|  | Estimate | Std. Error | t | P |  |  | Estimate | Std. Error | t | P |
| (Intercept) | 3.821 | 0.883 | 4.328 | 0.000 |  | (Intercept) | 3.363 | 1.150 | 2.925 | 0.007 |
| GroupME/CFS | 0.687 | 0.227 | 3.028 | **0.005** |  | GroupME/CFS | 1.148 | 0.354 | 3.246 | **0.003** |
| demog_age | -0.009 | 0.007 | -1.304 | 0.203 |  | demog_age | -0.002 | 0.010 | -0.194 | 0.848 |
| demog_sex | -0.453 | 0.223 | -2.028 | 0.052 |  | demog_sex | 0.033 | 0.319 | 0.105 | 0.917 |
| demog_bmi | 0.003 | 0.028 | 0.099 | 0.922 |  | demog_bmi | -0.037 | 0.044 | -0.845 | 0.406 |
| demog_income | 0.015 | 0.007 | 2.256 | **0.032** |  | demog_income | 0.001 | 0.010 | 0.105 | 0.917 |
| demog_work_ondisab | -0.384 | 0.322 | -1.191 | 0.243 |  | demog_work_ondisab | -0.175 | 0.475 | -0.368 | 0.716 |
| texturerunny | 0.782 | 0.626 | 1.248 | 0.222 |  | texturerunny | 1.586 | 0.724 | 2.192 | **0.037** |
| texturesoft | 0.077 | 0.231 | 0.333 | 0.742 |  | texturesoft | 0.583 | 0.365 | 1.600 | 0.121 |
| processing_time | -0.208 | 0.071 | -2.927 | **0.007** |  | processing_time | 0.127 | 0.112 | 1.139 | 0.265 |
|  |  |  |  |  |  |  |  |  |  |  |
| Adjusted R-squared: 0.4359 | | | | |  | Adjusted R-squared: 0.2612 | | | | |
| F-statistic: 4.177 on 9 and 28 DF, p-value: 0.001679 | | | | |  | F-statistic: 2.414 on 9 and 27 DF, p-value: 0.03704 | | | | |
| Indoleacetate |  |  |  |  |  | 4-hydroxyphenyl**acetate** | |  |  |  |
|  | Estimate | Std. Error | t | P |  |  | Estimate | Std. Error | t | P |
| (Intercept) | 2.494 | 1.227 | 2.033 | 0.051 |  | (Intercept) | 1.973 | 0.863 | 2.287 | 0.030 |
| GroupME/CFS | 0.821 | 0.387 | 2.123 | **0.042** |  | GroupME/CFS | 0.980 | 0.292 | 3.363 | **0.002** |
| demog_age | -0.030 | 0.011 | -2.630 | **0.014** |  | demog_age | -0.006 | 0.008 | -0.677 | 0.504 |
| demog_sex | -0.328 | 0.335 | -0.979 | 0.336 |  | demog_sex | -0.457 | 0.249 | -1.833 | 0.078 |
| demog_bmi | 0.053 | 0.042 | 1.255 | 0.220 |  | demog_bmi | 0.043 | 0.030 | 1.424 | 0.166 |
| demog_income | -0.002 | 0.011 | -0.203 | 0.840 |  | demog_income | 0.004 | 0.008 | 0.453 | 0.654 |
| demog_work_ondisab | 0.288 | 0.525 | 0.548 | 0.588 |  | demog_work_ondisab | -0.051 | 0.404 | -0.126 | 0.900 |
| texturerunny | -0.527 | 0.773 | -0.682 | 0.501 |  | texturerunny | -0.163 | 0.741 | -0.221 | 0.827 |
| texturesoft | 0.034 | 0.361 | 0.095 | **0.925** |  | texturesoft | 0.054 | 0.255 | 0.210 | 0.835 |
| processing_time | -0.375 | 0.110 | -3.418 | 0.002 |  | processing_time | -0.260 | 0.075 | -3.461 | **0.002** |
|  |  |  |  |  |  |  |  |  |  |  |
| Adjusted R-squared: 0.3526 | | | | |  | Adjusted R-squared: 0.4408 | | | | |
| F-statistic: 3.3 on 9 and 29 DF, p-value: 0.006897 | | | | |  | F-statistic: 4.153 on 9 and 27 DF, p-value: 0.001901 | | | | |
| 4-hydroxyphenyl**acrylate** | |  |  |  |  | phenyl pyruvate** |  |  |  |  |
|  | Estimate | Std. Error | t | P |  |  | Estimate | Std. Error | t | P |
| (Intercept) | -1.394 | 0.951 | -1.465 | 0.154 |  | (Intercept) | -0.621 | 1.671 | -0.372 | 0.713 |
| GroupME/CFS | 0.698 | 0.268 | 2.602 | **0.015** |  | GroupME/CFS | 0.904 | 0.419 | 2.155 | **0.040** |
| demog_age | -0.009 | 0.007 | -1.282 | 0.211 |  | demog_age | 0.000 | 0.013 | 0.031 | 0.975 |
| demog_sex | -0.673 | 0.234 | -2.871 | **0.008** |  | demog_sex | -0.873 | 0.435 | -2.007 | 0.055 |
| demog_bmi | 0.056 | 0.033 | 1.686 | 0.103 |  | demog_bmi | 0.070 | 0.051 | 1.365 | 0.183 |
| demog_income | -0.004 | 0.008 | -0.550 | 0.586 |  | demog_income | -0.014 | 0.012 | -1.159 | 0.256 |
| demog_work_ondisab | -0.309 | 0.363 | -0.853 | 0.401 |  | demog_work_ondisab | -0.358 | 0.599 | -0.597 | 0.555 |
| texturerunny | 0.326 | 0.533 | 0.612 | 0.546 |  | texturerunny | 1.639 | 1.258 | 1.303 | 0.203 |
| texturesoft | -0.104 | 0.272 | -0.381 | 0.706 |  | texturesoft | 0.153 | 0.448 | 0.341 | 0.736 |
| processing_time | -0.107 | 0.077 | -1.385 | 0.177 |  | processing_time | -0.158 | 0.127 | -1.245 | 0.224 |
|  |  |  |  |  |  |  |  |  |  |  |
| Adjusted R-squared: 0.3298 | | | | |  | Adjusted R-squared: 0.2659 | | | | |
| F-statistic: 3.023 on 9 and 28 DF, p-value: 0.01187 | | | | |  | F-statistic: 2.489 on 9 and 28 DF, p-value: 0.03127 | | | | |
| Phenol** |  |  |  |  |  | 4-hydroxy-phenylpropionate | |  |  |  |
|  | Estimate | Std. Error | t | P |  |  | Estimate | Std. Error | t | P |
| (Intercept) | -2.806 | 1.243 | -2.258 | 0.032 |  | (Intercept) | -0.627 | 1.843 | -0.340 | 0.736 |
| GroupME/CFS | 1.170 | 0.393 | 2.974 | **0.006** |  | GroupME/CFS | 2.196 | 0.516 | 4.254 | **0.000** |
| demog_age | -0.005 | 0.012 | -0.413 | 0.683 |  | demog_age | -0.024 | 0.015 | -1.610 | 0.119 |
| demog_sex | -0.542 | 0.356 | -1.525 | 0.139 |  | demog_sex | -0.224 | 0.465 | -0.482 | 0.634 |
| demog_bmi | 0.071 | 0.043 | 1.648 | 0.111 |  | demog_bmi | 0.069 | 0.062 | 1.101 | 0.281 |
| demog_income | -0.009 | 0.011 | -0.837 | 0.410 |  | demog_income | -0.012 | 0.015 | -0.808 | 0.426 |
| demog_work_ondisab | -1.054 | 0.541 | -1.948 | 0.062 |  | demog_work_ondisab | -1.053 | 0.701 | -1.501 | 0.145 |
| texturerunny | 2.040 | 1.054 | 1.935 | 0.063 |  | texturerunny | 1.165 | 1.018 | 1.145 | 0.262 |
| texturesoft | 0.637 | 0.364 | 1.749 | 0.092 |  | texturesoft | -0.103 | 0.515 | -0.201 | 0.842 |
| processing_time | 0.279 | 0.113 | 2.459 | **0.021** |  | processing_time | 0.165 | 0.145 | 1.135 | 0.266 |
|  |  |  |  |  |  |  |  |  |  |  |
| Adjusted R-squared: 0.364 | | | | |  | Adjusted R-squared: 0.3772 | | | | |
| F-statistic: 3.289 on 9 and 27 DF, p-value: 0.007861 | | | | |  | F-statistic: 3.423 on 9 and 27 DF, p-value: 0.006265 | | | | |
| Phenyllactate |  |  |  |  |  |  |  |  |  |  |
|  | Estimate | Std. Error | t | P |  |  |  |  |  |  |
| (Intercept) | 0.819 | 1.274 | 0.643 | 0.526 |  |  |  |  |  |  |
| GroupME/CFS | 0.897 | 0.338 | 2.650 | **0.013** |  |  |  |  |  |  |
| demog_age | -0.012 | 0.010 | -1.202 | 0.240 |  |  |  |  |  |  |
| demog_sex | -0.466 | 0.340 | -1.372 | 0.181 |  |  |  |  |  |  |
| demog_bmi | 0.016 | 0.040 | 0.410 | 0.685 |  |  |  |  |  |  |
| demog_income | 0.007 | 0.010 | 0.661 | 0.514 |  |  |  |  |  |  |
| demog_work_ondisab | -1.227 | 0.483 | -2.539 | **0.017** |  |  |  |  |  |  |
| texturesoft | -0.178 | 0.350 | -0.509 | 0.615 |  |  |  |  |  |  |
| processing_time | -0.001 | 0.100 | -0.009 | 0.993 |  |  |  |  |  |  |
|  |  |  |  |  |  |  |  |  |  |  |
|  |  |  |  |  |  |  |  |  |  |  |
| Adjusted R-squared: 0.222 | | | | |  |  |  |  |  |  |
| F-statistic: 2.248 on 8 and 27 DF, p-value: 0.05523 | | | | |  |  |  |  |  |  |
|  |  |  |  |  |  |  |  |  |  |  |
|  |  |  |  |  |  |  |  |  |  |  |

| Simplified Models* |  |  |  |  |  |  |  |  |  |  |
| --- | --- | --- | --- | --- | --- | --- | --- | --- | --- | --- |
| Tryptophan** |  |  |  |  |  | Indole** |  |  |  |  |
|  | Estimate | Std. Error | t | P |  |  | Estimate | Std. Error | t | P |
| (Intercept) | 3.050 | 0.254 | 11.983 | 0.000 |  | (Intercept) | 2.993 | 0.274 | 10.930 | 0.000 |
| GroupME/CFS | 0.609 | 0.202 | 3.009 | **0.005** |  | GroupME/CFS | 1.123 | 0.271 | 4.141 | **0.000** |
| demog_income | 0.019 | 0.006 | 2.878 | **0.007** |  | texturerunny | 1.265 | 0.630 | 2.009 | **0.053** |
| processing_time | -0.188 | 0.064 | -2.961 | **0.006** |  | texturesoft | 0.327 | 0.294 | 1.113 | 0.274 |
|  |  |  |  |  |  |  |  |  |  |  |
| Adjusted R-squared: 0.3844 | | | | |  | Adjusted R-squared: 0.3402 | | | | |
| F-statistic: 8.702 on 3 and 34 DF, p-value: 0.0002015 | | | | |  | F-statistic: 7.187 on 3 and 33 DF, p-value: 0.0007661 | | | | |
| Indoleacetate** |  |  |  |  |  | 4-hydroxyphenyl**acetate**** | |  |  |  |
|  | Estimate | Std. Error | t | P |  |  | Estimate | Std. Error | t | P |
| (Intercept) | 3.321 | 0.724 | 4.589 | 0.000 |  | (Intercept) | 2.423 | 0.284 | 8.539 | 0.000 |
| GroupME/CFS | 0.997 | 0.308 | 3.235 | **0.003** |  | GroupME/CFS | 1.052 | 0.225 | 4.675 | **0.000** |
| demog_age | -0.025 | 0.010 | -2.462 | **0.019** |  | processing_time | -0.245 | 0.068 | -3.606 | **0.001** |
| processing_time | -0.380 | 0.098 | -3.877 | **0.000** |  |  |  |  |  |  |
|  |  |  |  |  |  |  |  |  |  |  |
| Adjusted R-squared: 0.404 | | | | |  | Adjusted R-squared: 0.4549 | | | | |
| F-statistic: 9.587 on 3 and 35 DF, p-value: 9.233e-05 | | | | |  | F-statistic: 16.02 on 2 and 34 DF, p-value: 1.254e-05 | | | | |
| 4-hydroxyphenyl**acrylate**** | |  |  |  |  | phenyl pyruvate |  |  |  |  |
|  | Estimate | Std. Error | t | P |  |  | Estimate | Std. Error | t | P |
| (Intercept) | -1.083 | 0.206 | -5.251 | 0.000 |  | (Intercept) | -0.046 | 0.274 | -0.169 | 0.867 |
| GroupME/CFS | 0.691 | 0.214 | 3.220 | **0.003** |  | GroupME/CFS | 0.845 | 0.387 | 2.183 | **0.036** |
| demog_sex | -0.585 | 0.222 | -2.633 | **0.013** |  |  |  |  |  |  |
|  |  |  |  |  |  |  |  |  |  |  |
|  |  |  |  |  |  |  |  |  |  |  |
| Adjusted R-squared: 0.3021 | | | | |  | Adjusted R-squared: 0.18 | | | | |
| F-statistic: 9.007 on 2 and 35 DF, p-value: 0.0006989 | | | | |  | F-statistic: 5.06 on 2 and 35 DF, p-value: 0.01174 | | | | |
| Phenol |  |  |  |  |  | 4-hydroxy-phenylpropionate** | |  |  |  |
|  | Estimate | Std. Error | t | P |  |  | Estimate | Std. Error | t | P |
| (Intercept) | -0.938 | 0.482 | -1.947 | 0.060 |  | (Intercept) | 0.165 | 0.284 | 0.581 | 0.565 |
| GroupME/CFS | 0.925 | 0.366 | 2.526 | **0.016** |  | GroupME/CFS | 1.933 | 0.407 | 4.748 | **0.000** |
| processing_time | 0.174 | 0.116 | 1.501 | **0.143** |  |  |  |  |  |  |
|  |  |  |  |  |  |  |  |  |  |  |
|  |  |  |  |  |  |  |  |  |  |  |
| Adjusted R-squared: 0.1717 | | | | |  | Adjusted R-squared: 0.3744 | | | | |
| F-statistic: 4.731 on 2 and 34 DF, p-value: 0.01539 | | | | |  | F-statistic: 22.55 on 1 and 35 DF, p-value: 3.424e-05 | | | | |
| Phenyllactate** |  |  |  |  |  |  |  |  |  |  |
|  | Estimate | Std. Error | t | P |  |  |  |  |  |  |
| (Intercept) | 0.137 | 0.189 | 0.725 | 0.474 |  |  |  |  |  |  |
| GroupME/CFS | 1.039 | 0.303 | 3.429 | **0.002** |  |  |  |  |  |  |
| demog_work_ondisab | -1.174 | 0.438 | -2.683 | **0.011** |  |  |  |  |  |  |
| --- |  |  |  |  |  |  |  |  |  |  |
|  |  |  |  |  |  |  |  |  |  |  |
| Adjusted R-squared: 0.2488 | | | | |  |  |  |  |  |  |
| F-statistic: 6.797 on 2 and 33 DF, p-value: 0.003372 | | | | |  |  |  |  |  |  |
|  |  |  |  |  |  |  |  |  |  |  |
|  |  |  |  |  |  |  |  |  |  |  |
| *Simplified models generated through backwards elimination of non-significant (P>0.05) predictors from full models | | | | | | | | | | |
| ** best model for that metabolite by AIC | | | | | | | | | | |
