# Supplementary material for: Tryptophan Metabolism and Aryl‐Hydrocarbon Receptor Agonists in the Gut Microbiome of People With Myalgic Encephalomyelitis/Chronic Fatigue Syndrome
Source: Microbiologyopen. 2026 Jun 22;15(3):e70333. doi: 10.1002/mbo3.70333 (PMC13284739; doi:10.1002/mbo3.70333)
Supplement: Supplementary file 4 — Table A4: Alpha diversity models with Trp correlated subcommunity. [file MBO3-15-e70333-s005.docx]

| DISEASE (MECFS vs Controls), DEMOGRAPHICS, STOOL PROPERTIES MODELS | | | | | |  | |  | |  | |
| --- | --- | --- | --- | --- | --- | --- | --- | --- | --- | --- | --- |
| with TRP-CORRELATED MICROBIOME SUBCOMMUNITY | | | |  |  |  | |  | |  | |
|  |  |  |  |  |  |  | |  | |  | |
| Full Models | Shannon index | |  |  | Faith's PD (log transformed) | | | | |  | |
|  | Estimate | Std. Error | t | P | Estimate | Std. Error | | t | | P | |
| (Intercept) | 4.801 | 0.372 | 12.908 | <2.00E-16 | 1.744 | 0.251 | | 6.952 | | 0.000 | |
| Disease(MECFS) | -0.354 | 0.107 | -3.304 | **0.002** | -0.061 | 0.072 | | -0.851 | | 0.400 | |
| demog_age | 0.011 | 0.003 | 3.289 | **0.002** | 0.005 | 0.002 | | 2.031 | | **0.048** | |
| demog_sex | 0.145 | 0.098 | 1.481 | 0.146 | 0.037 | 0.066 | | 0.553 | | 0.583 | |
| demog_bmi | -0.009 | 0.014 | -0.683 | 0.498 | 0.001 | 0.009 | | 0.140 | | 0.890 | |
| demog_income | 0.009 | 0.004 | 2.408 | **0.020** | 0.004 | 0.003 | | 1.769 | | 0.084 | |
| demog_work_ondisab | 0.057 | 0.163 | 0.348 | 0.730 | 0.003 | 0.110 | | 0.028 | | 0.978 | |
| texturerunny | -0.193 | 0.265 | -0.729 | 0.470 | 0.049 | 0.179 | | 0.273 | | 0.786 | |
| texturesoft | 0.043 | 0.110 | 0.390 | 0.698 | 0.039 | 0.074 | | 0.528 | | 0.600 | |
| processing_time | 0.034 | 0.030 | 1.127 | 0.266 | 0.013 | 0.020 | | 0.631 | | 0.531 | |
| --- | p-value: 0.005017 | |  |  | p-value: 0.5658 | | |  | |  | |
|  | Adjusted R-squared: 0.2684 | | |  | Adjusted R-squared: -0.02412 | | | | |  | |
|  | F-statistic: 3.161 on 9 and 44 DF | | |  | F-statistic: 0.8613 on 9 and 44 DF | | | | |  | |
|  |  |  |  |  |  |  | |  | |  | |
| Full Models | Pielou evenness | |  |  | Dominance | | |  | |  | |
|  | Estimate | Std. Error | t | P | Estimate | Std. Error | | t | | P | |
| (Intercept) | 0.928 | 0.040 | 23.060 | <2e-16 | 0.037 | 0.013 | | 2.938 | | **0.005** | |
| Disease(MECFS) | -0.030 | 0.012 | -2.571 | **0.014** | 0.011 | 0.004 | | 3.090 | | **0.003** | |
| demog_age | 0.000 | 0.000 | 0.151 | 0.881 | 0.000 | 0.000 | | -2.035 | | **0.048** | |
| demog_sex | 0.011 | 0.011 | 1.053 | 0.298 | -0.005 | 0.003 | | -1.645 | | 0.107 | |
| demog_bmi | -0.001 | 0.002 | -0.416 | 0.679 | 0.000 | 0.000 | | 0.985 | | 0.330 | |
| demog_income | 0.000 | 0.000 | -1.071 | 0.290 | 0.000 | 0.000 | | -1.898 | | 0.064 | |
| demog_work_ondisab | 0.010 | 0.018 | 0.540 | 0.592 | -0.004 | 0.006 | | -0.697 | | 0.489 | |
| texturerunny | -0.022 | 0.029 | -0.752 | 0.456 | 0.006 | 0.009 | | 0.647 | | 0.521 | |
| texturesoft | -0.002 | 0.012 | -0.136 | 0.892 | -0.001 | 0.004 | | -0.202 | | 0.841 | |
| processing_time | -0.005 | 0.003 | -1.501 | 0.140 | 0.000 | 0.001 | | 0.380 | | 0.706 | |
|  | p-value: 0.1047 | |  |  | p-value: 0.04081 | | |  | |  | |
|  | Adjusted R-squared: 0.1138 | | |  | Adjusted R-squared: 0.1686 | | | | |  | |
|  | F-statistic: 1.756 on 9 and 44 DF | | |  | F-statistic: 2.194 on 9 and 44 DF | | | | |  | |
|  |  |  |  |  |  |  | |  | |  | |
|  |  |  |  |  |  |  | |  | |  | |
|  |  |  |  |  |  |  | |  | |  | |
| Simplified Models* | Shannon index** | |  |  | Faith's PD |  | |  | |  | |
|  | Estimate | Std. Error | t | P | Estimate | Std. Error | | t | | P | |
| (Intercept) | 4.865 | 0.167 | 29.078 | <2.00E-16 | no simplified model constructed | | | | |  | |
| Meets_criteriaYes | -0.342 | 0.093 | -3.692 | **0.001** |  |  | |  | |  | |
| demog_age | 0.009 | 0.003 | 3.103 | **0.003** |  |  | |  | |  | |
| demog_income | 0.009 | 0.003 | 2.719 | **0.009** |  |  | |  | |  | |
|  | p-value: 0.000125 | |  |  |  |  | |  | |  | |
|  | Adjusted R-squared: 0.2958 | | |  |  |  | |  | |  | |
|  | F-statistic: 8.419 on 3 and 50 DF | | |  |  |  | |  | |  | |
|  |  |  |  |  |  |  | |  | |  | |
| Simplified Models* | Pielou evenness** | |  |  | Dominance** | | |  | |  | |
|  | Estimate | Std. Error | t | P | Estimate | Std. Error | | t | | P | |
| (Intercept) | 0.898 | 0.007 | 132.735 | <2.00E-16 | 0.043 | 0.006 | | 7.463 | | 0.000 | |
| Meets_criteriaYes | -0.029 | 0.010 | -2.902 | **0.005** | 0.009 | 0.003 | | 2.746 | | **0.008** | |
| demog_age |  |  |  |  | 0.000 | | 0.000 | | -1.677 | | **0.099** |
| demog_income |  |  |  |  |  |  | |  | |  | |
|  | p-value: 0.005357 | |  |  | p-value: 0.009727 | | |  | |  | |
|  | Adjusted R-squared: 0.1189 | | |  | Adjusted R-squared: 0.1287 | | | | |  | |
|  | F-statistic: 8.421 on 1 and 54 DF | | |  | F-statistic: 5.062 on 2 and 53 DF | | | | |  | |
