# Supplementary material for: Tryptophan Metabolism and Aryl‐Hydrocarbon Receptor Agonists in the Gut Microbiome of People With Myalgic Encephalomyelitis/Chronic Fatigue Syndrome
Source: Microbiologyopen. 2026 Jun 22;15(3):e70333. doi: 10.1002/mbo3.70333 (PMC13284739; doi:10.1002/mbo3.70333)
Supplement: Supplementary file 5 — Table A5: Beta diversity models with Trp correlated subcommunity. [file MBO3-15-e70333-s004.docx]

|  | |  |  |  |  |  | |  |  |
| --- | --- | --- | --- | --- | --- | --- | --- | --- | --- |
| DISEASE (MECFS vs Control) DEMOGRAPHICS, STOOL PROPERTIES MODELS with TRP CORRELATED SUBCOMMUNITY | | | | | | | | |  |
|  | |  |  |  |  |  | |  |  |
| Full Models | | Bray Curtis |  |  |  |  | |  |  |
|  | |  | Df | SumOfSqs | R2 | pseudo-F | | Pr(>F) |  |
|  | | Disease | 1 | 0.491 | 0.030 | 1.939 | | **0.007** |  |
|  | | age | 1 | 0.448 | 0.028 | 1.767 | | **0.013** |  |
|  | | sex | 1 | 0.269 | 0.017 | 1.061 | | 0.351 |  |
|  | | bmi | 1 | 0.262 | 0.016 | 1.034 | | 0.409 |  |
|  | | income | 1 | 0.387 | 0.024 | 1.528 | | 0.097 |  |
|  | | disab | 1 | 0.146 | 0.009 | 0.575 | | 0.964 |  |
|  | | texture | 2 | 1.165 | 0.072 | 2.300 | | **0.001** |  |
|  | | storage | 1 | 0.415 | 0.026 | 1.638 | | **0.030** |  |
|  | | Residual | 49 | 12.413 | 0.767 |  | |  |  |
|  | | Total | 58 | 16.177 | 1.000 |  | |  |  |
|  | |  |  |  |  |  | |  |  |
|  | | Jaccard |  |  |  |  | |  |  |
|  | |  | Df | SumOfSqs | R2 | pseudo-F | | Pr(>F) |  |
|  | | Disease | 1 | 0.471 | 0.025 | 1.548 | | **0.009** |  |
|  | | age | 1 | 0.506 | 0.027 | 1.664 | | **0.003** |  |
|  | | sex | 1 | 0.360 | 0.019 | 1.184 | | 0.135 |  |
|  | | bmi | 1 | 0.340 | 0.018 | 1.120 | | 0.234 |  |
|  | | income | 1 | 0.364 | 0.020 | 1.199 | | 0.145 |  |
|  | | disab | 1 | 0.258 | 0.014 | 0.850 | | 0.804 |  |
|  | | texture | 2 | 0.907 | 0.049 | 1.493 | | **0.003** |  |
|  | | storage | 1 | 0.479 | 0.026 | 1.576 | | **0.005** |  |
|  | | Residual | 49 | 14.894 | 0.802 |  | |  |  |
|  | | Total | 58 | 18.576 | 1.000 |  | |  |  |
|  | |  |  |  |  |  | |  |  |
|  | | Weighted UniFrac | |  |  |  | |  |  |
|  | |  | Df | SumOfSqs | R2 | pseudo-F | | Pr(>F) |  |
|  | | Disease | 1 | 0.097 | 0.027 | 1.755 | | 0.092 |  |
|  | | age | 1 | 0.100 | 0.027 | 1.802 | | 0.097 |  |
|  | | sex | 1 | 0.052 | 0.014 | 0.947 | | 0.490 |  |
|  | | bmi | 1 | 0.083 | 0.023 | 1.506 | | 0.171 |  |
|  | | income | 1 | 0.018 | 0.005 | 0.329 | | 0.914 |  |
|  | | disab | 1 | 0.033 | 0.009 | 0.598 | | 0.742 |  |
|  | | texture | 2 | 0.382 | 0.105 | 3.460 | | **0.002** |  |
|  | | storage | 1 | 0.054 | 0.015 | 0.972 | | 0.439 |  |
|  | | Residual | 49 | 2.707 | 0.746 |  | |  |  |
|  | | Total | 58 | 3.628 | 1.000 |  | |  |  |
|  | |  |  |  |  |  | |  |  |
|  | | Unweighted UniFrac | |  |  |  | |  |  |
|  | |  | Df | SumOfSqs | R2 | pseudo-F | | Pr(>F) |  |
|  | | Disease | 1 | 0.215 | 0.028 | 1.659 | | **0.043** |  |
|  | | age | 1 | 0.195 | 0.025 | 1.508 | | 0.083 |  |
|  | | sex | 1 | 0.107 | 0.014 | 0.827 | | 0.663 |  |
|  | | bmi | 1 | 0.106 | 0.014 | 0.819 | | 0.666 |  |
|  | | income | 1 | 0.165 | 0.021 | 1.275 | | 0.166 |  |
|  | | disab | 1 | 0.107 | 0.014 | 0.826 | | 0.644 |  |
|  | | texture | 2 | 0.295 | 0.038 | 1.139 | | 0.248 |  |
|  | | storage | 1 | 0.209 | 0.027 | 1.616 | | 0.062 |  |
|  | | Residual | 49 | 6.351 | 0.816 |  | |  |  |
|  | | Total | 58 | 7.785 | 1.000 |  | |  |  |
| Simplified Models* | Bray Curtis | |  |  |  | |  |  | |
|  |  | | Df | SumOfSqs | R2 | | pseudo-F | Pr(>F) | |
|  | Disease | | 1 | 0.556 | 0.033 | | 2.219 | 0.002 | |
|  | age | | 1 | 0.419 | 0.025 | | 1.672 | 0.029 | |
|  | texture | | 2 | 1.207 | 0.072 | | 2.408 | 0.001 | |
|  | storage | | 1 | 0.486 | 0.029 | | 1.939 | 0.006 | |
|  | Residual | | 56 | 14.042 | 0.840 | |  |  | |
|  | Total | | 61 | 16.709 | 1.000 | |  |  | |
|  |  | |  |  |  | |  |  | |
|  | Jaccard | |  |  |  | |  |  | |
|  |  | | Df | SumOfSqs | R2 | | pseudo-F | Pr(>F) | |
|  | Disease | | 1 | 0.549 | 0.028 | | 1.807 | 0.002 | |
|  | age | | 1 | 0.454 | 0.023 | | 1.493 | 0.014 | |
|  | texture | | 2 | 0.896 | 0.046 | | 1.474 | 0.002 | |
|  | storage | | 1 | 0.470 | 0.024 | | 1.545 | 0.009 | |
|  | Residual | | 56 | 17.023 | 0.879 | |  |  | |
|  | Total | | 61 | 19.366 | 1.000 | |  |  | |
|  |  | |  |  |  | |  |  | |
|  | Weighted UniFrac | |  |  |  | |  |  | |
|  |  | | Df | SumOfSqs | R2 | | pseudo-F | Pr(>F) | |
|  |  | |  |  |  | |  |  | |
|  | simplified model not constructed | | | |  | |  |  | |
|  |  | |  |  |  | |  |  | |
|  |  | |  |  |  | |  |  | |
|  |  | |  |  |  | |  |  | |
|  |  | |  |  |  | |  |  | |
|  |  | |  |  |  | |  |  | |
|  | Unweighted UniFrac | | |  |  | |  |  | |
|  |  | | Df | SumOfSqs | R2 | | pseudo-F | Pr(>F) | |
|  | Disease | | 1 | 0.249 | 0.029 | | 1.824 | 0.025 | |
|  | Residual | | 61 | 8.311 | 0.971 | |  |  | |
|  | Total | | 62 | 8.559 | 1.000 | |  |  | |
|  |  | |  |  |  | |  |  | |
|  |  | |  |  |  | |  |  | |
|  |  | |  |  |  | |  |  | |
|  |  | |  |  |  | |  |  | |
|  | * constructed by backwards elimination of nonsignificant (P>0.05) variables from full model | | | | | | | | |
