# Supplementary material for: Tryptophan Metabolism and Aryl‐Hydrocarbon Receptor Agonists in the Gut Microbiome of People With Myalgic Encephalomyelitis/Chronic Fatigue Syndrome
Source: Microbiologyopen. 2026 Jun 22;15(3):e70333. doi: 10.1002/mbo3.70333 (PMC13284739; doi:10.1002/mbo3.70333)
Supplement: Supplementary file 6 — Table A6: Alpha diversity symptom models. [file MBO3-15-e70333-s006.docx]

|  |  |  |  |  |
| --- | --- | --- | --- | --- |
| SYMPTOMS MODELS with full microbiome community | | |  |  |
|  |  |  |  |  |
|  | Shannon index | |  |  |
|  | Estimate | Std. Error | t | P |
| (Intercept) | 6.385 | 0.267 | 23.898 | <2e-16 |
| Meets_criteriaYes | -0.307 | 0.131 | -2.341 | **0.023** |
| sa_full_gut_ibs0 | 0.167 | 0.252 | 0.664 | 0.510 |
| sa_full_gut_ibs1 | 0.239 | 0.282 | 0.847 | 0.401 |
| neurocog_symptomsYes | 0.043 | 0.141 | 0.306 | 0.761 |
| texturerunny | -0.140 | 0.257 | -0.545 | 0.588 |
| texturesoft | 0.071 | 0.097 | 0.734 | 0.466 |
| processing_time | 0.040 | 0.028 | 1.439 | 0.157 |
|  |  |  |  |  |
|  | Adjusted R-squared: 0.07585 | | |  |
|  | F-statistic: 1.657 on 7 and 49 DF, p-value: 0.142 | | | |
|  |  |  |  |  |
|  | Faith's PD (log transformed) | | |  |
|  | Estimate | Std. Error | t | P |
| (Intercept) | 2.526 | 0.175 | 14.472 | <2e-16 |
| Meets_criteriaYes | -0.108 | 0.086 | -1.264 | 0.212 |
| sa_full_gut_ibs0 | 0.152 | 0.165 | 0.926 | 0.359 |
| sa_full_gut_ibs1 | 0.173 | 0.184 | 0.938 | 0.353 |
| neurocog_symptomsYes | 0.044 | 0.092 | 0.474 | 0.638 |
| texturerunny | 0.014 | 0.168 | 0.085 | 0.932 |
| texturesoft | 0.005 | 0.063 | 0.083 | 0.934 |
| processing_time | 0.019 | 0.018 | 1.019 | 0.313 |
|  |  |  |  |  |
|  | Adjusted R-squared: -0.05327 | | |  |
|  | F-statistic: 0.5954 on 7 and 49 DF, p-value: 0.7565 | | | |
|  |  |  |  |  |
|  |  |  |  |  |
|  | Pielou evenness | |  |  |
|  | Estimate | Std. Error | t | P |
| (Intercept) | 0.919 | 0.022 | 41.755 | <2e-16 |
| Meets_criteriaYes | -0.019 | 0.011 | -1.785 | 0.080 |
| sa_full_gut_ibs0 | -0.002 | 0.021 | -0.082 | 0.935 |
| sa_full_gut_ibs1 | 0.001 | 0.023 | 0.030 | 0.976 |
| neurocog_symptomsYes | -0.009 | 0.012 | -0.761 | 0.450 |
| texturerunny | -0.010 | 0.021 | -0.488 | 0.628 |
| texturesoft | 0.002 | 0.008 | 0.210 | 0.835 |
| processing_time | -0.003 | 0.002 | -1.340 | 0.186 |
|  |  |  |  |  |
|  | Adjusted R-squared: 0.1153 | | |  |
|  | F-statistic: 2.043 on 7 and 49 DF, p-value: 0.06825 | | | |
|  |  |  |  |  |
|  | Dominance |  |  |  |
|  | Estimate | Std. Error | t | P |
| (Intercept) | 0.018 | 0.003 | 5.450 | 0.000 |
| Meets_criteriaYes | 0.004 | 0.002 | 2.312 | **0.025** |
| sa_full_gut_ibs0 | -0.003 | 0.003 | -1.049 | 0.299 |
| sa_full_gut_ibs1 | -0.004 | 0.004 | -1.138 | 0.261 |
| neurocog_symptomsYes | 0.000 | 0.002 | 0.153 | 0.879 |
| texturerunny | 0.002 | 0.003 | 0.554 | 0.582 |
| texturesoft | -0.001 | 0.001 | -0.846 | 0.402 |
| processing_time | 0.000 | 0.000 | -0.603 | 0.549 |
|  |  |  |  |  |
|  | Adjusted R-squared: 0.08765 | | |  |
|  | F-statistic: 1.769 on 7 and 49 DF, p-value: 0.1152 | | | |
