# Supplementary material for: Tryptophan Metabolism and Aryl‐Hydrocarbon Receptor Agonists in the Gut Microbiome of People With Myalgic Encephalomyelitis/Chronic Fatigue Syndrome
Source: Microbiologyopen. 2026 Jun 22;15(3):e70333. doi: 10.1002/mbo3.70333 (PMC13284739; doi:10.1002/mbo3.70333)
Supplement: Supplementary file 7 — Table A7: Beta diversity symptom models. [file MBO3-15-e70333-s002.docx]

|  |  |  |  |  |  |  |
| --- | --- | --- | --- | --- | --- | --- |
| SYMPTOMS MODELS with WHOLE MICROBIOME COMMUNITY | | | |  |  |  |
|  |  |  |  |  |  |  |
|  | Bray Curtis |  |  |  |  |  |
|  |  | Df | SumOfSqs | R2 | pseudo-F | Pr(>F) |
|  | Disease | 1 | 0.410 | 0.027 | 1.571 | **0.009** |
|  | neurocog_symptoms | 1 | 0.301 | 0.020 | 1.153 | 0.192 |
|  | sa_full_gut_ibs | 1 | 0.310 | 0.020 | 1.188 | 0.154 |
|  | texture | 2 | 0.833 | 0.054 | 1.596 | **0.001** |
|  | storage | 1 | 0.387 | 0.025 | 1.481 | **0.017** |
|  | Residual | 50 | 13.048 | 0.852 |  |  |
|  | Total | 56 | 15.319 | 1.000 |  |  |
|  |  |  |  |  |  |  |
|  |  |  |  |  |  |  |
|  | Jaccard |  |  |  |  |  |
|  |  | Df | SumOfSqs | R2 | pseudo-F | Pr(>F) |
|  | Disease | 1 | 0.449 | 0.022 | 1.273 | **0.007** |
|  | neurocog_symptoms | 1 | 0.352 | 0.018 | 0.997 | 0.468 |
|  | sa_full_gut_ibs | 1 | 0.400 | 0.020 | 1.133 | 0.089 |
|  | texture | 2 | 0.761 | 0.038 | 1.078 | 0.127 |
|  | storage | 1 | 0.425 | 0.021 | 1.205 | **0.026** |
|  | Residual | 50 | 17.635 | 0.879 |  |  |
|  | Total | 56 | 20.059 | 1.000 |  |  |
|  |  |  |  |  |  |  |
|  |  |  |  |  |  |  |
|  | Weighted UniFrac |  |  |  |  |  |
|  |  | Df | SumOfSqs | R2 | pseudo-F | Pr(>F) |
|  | Disease | 1 | 0.112 | 0.058 | 3.537 | **0.004** |
|  | neurocog_symptoms | 1 | 0.047 | 0.025 | 1.487 | 0.158 |
|  | sa_full_gut_ibs | 1 | 0.019 | 0.010 | 0.602 | 0.733 |
|  | texture | 2 | 0.081 | 0.043 | 1.286 | 0.192 |
|  | storage | 1 | 0.073 | 0.039 | 2.329 | **0.028** |
|  | Residual | 50 | 1.578 | 0.827 |  |  |
|  | Total | 56 | 1.909 | 1.000 |  |  |
|  |  |  |  |  |  |  |
|  |  |  |  |  |  |  |
|  | Unweighted UniFrac | |  |  |  |  |
|  |  | Df | SumOfSqs | R2 | pseudo-F | Pr(>F) |
|  | Disease | 1 | 0.295 | 0.038 | 2.186 | **0.004** |
|  | neurocog_symptoms | 1 | 0.200 | 0.025 | 1.478 | 0.052 |
|  | sa_full_gut_ibs | 1 | 0.154 | 0.020 | 1.139 | 0.226 |
|  | texture | 2 | 0.257 | 0.033 | 0.951 | 0.558 |
|  | storage | 1 | 0.234 | 0.030 | 1.732 | **0.015** |
|  | Residual | 50 | 6.755 | 0.863 |  |  |
|  | Total | 56 | 7.832 | 1.000 |  |  |
|  |  |  |  |  |  |  |
